# Emotions and brain function are altered up to one month after a single high dose of psilocybin

**DOI:** 10.1038/s41598-020-59282-y

**Published:** 2020-02-10

**Authors:** Frederick S. Barrett, Manoj K. Doss, Nathan D. Sepeda, James J. Pekar, Roland R. Griffiths

**Affiliations:** 1 Center for Psychedelic and Consciousness Research, Department of Psychiatry and Behavioral Sciences, Johns Hopkins University School of Medicine, Baltimore, MD 21224 USA; 2 F.M. Kirby Research Center for Functional Brain Imaging, Kennedy Krieger Institute, Baltimore, MD 21205 USA; 3 Russell H. Morgan Department of Radiology and Radiological Science, Johns Hopkins University School of Medicine, Baltimore, MD 21205 USA; 4 Department of Neuroscience, Johns Hopkins University School of Medicine, Baltimore, MD 21224 USA

**Keywords:** Limbic system, Prefrontal cortex, Psychology

## Abstract

Psilocybin is a classic psychedelic compound that may have efficacy for the treatment of mood and substance use disorders. Acute psilocybin effects include reduced negative mood, increased positive mood, and reduced amygdala response to negative affective stimuli. However, no study has investigated the long-term, enduring impact of psilocybin on negative affect and associated brain function. Twelve healthy volunteers (7F/5M) completed an open-label pilot study including assessments 1-day before, 1-week after, and 1-month after receiving a 25 mg/70 kg dose of psilocybin to test the hypothesis that psilocybin administration leads to enduring changes in affect and neural correlates of affect. One-week post-psilocybin, negative affect and amygdala response to facial affect stimuli were reduced, whereas positive affect and dorsal lateral prefrontal and medial orbitofrontal cortex responses to emotionally-conflicting stimuli were increased. One-month post-psilocybin, negative affective and amygdala response to facial affect stimuli returned to baseline levels while positive affect remained elevated, and trait anxiety was reduced. Finally, the number of significant resting-state functional connections across the brain increased from baseline to 1-week and 1-month post-psilocybin. These preliminary findings suggest that psilocybin may increase emotional and brain plasticity, and the reported findings support the hypothesis that negative affect may be a therapeutic target for psilocybin.

## Introduction

Studies suggest that psilocybin, a classic psychedelic drug (serotonin 2A or 5-HT_2A_ receptor partial agonist), may have efficacy for the treatment of depression and anxiety^1–3^, tobacco use disorder^4,5^, and alcohol use disorder^6,7^. Reduction of clinical symptoms has been shown to last up to 3^3^, 6^1,2^, and 12^8^ months after 1 to 3 psilocybin administrations. Despite these promising advances, the neural and psychological mechanisms underlying the enduring therapeutic effects of psychedelic drugs are not well-understood. Two possibly interactive trans-diagnostic targets that may be affected by psilocybin are negative affect and brain network plasticity.

Increased negative affect, reduced positive affect, and hypersensitivity to negatively biased information are hallmarks of mood disorders^9–11^. Negative affect is also a core component of the cycle of addiction in which craving and withdrawal symptoms experienced after intoxication lead to preoccupation, anticipation, and re-administration of drugs of abuse^12^. The amygdala has been shown in clinical and preclinical models to track the salience of stimuli in the environment^13,14^ and is highly responsive to negative emotional stimuli^15–17^. Abnormally high amygdala reactivity to negative affective stimuli has been implicated in the pathophysiology of depression^18^. Areas within the anterior cingulate cortex (ACC) are understood to monitor cognitive conflict^19–22^, are involved in the appraisal and expression of negative emotion^22^, respond to distress levels associated with pain^23^ and negative social affect^24^, and have been implicated in negative rumination and depression^25^. Both amygdala and ACC dysfunction have been implicated in the pathophysiology of substance use disorders^12^ and have specifically been implicated in supporting aberrant negative affect in these disorders.

Psychedelic drugs have been shown to acutely reduce processing of negative affective stimuli^26^ while increasing positive mood in humans^27,28^. In behavioral paradigms, psychedelics have been shown to reduce sensitivity during encoding of fearful faces^29^, recognition of negative facial expressions^30^, and response to negative stimuli in an emotional inhibition task^27^. Functional magnetic resonance imaging (fMRI) studies have found that psilocybin acutely reduces amygdala activity and connectivity when viewing negative emotional facial expressions^28,31,32^. Psilocybin has also been found to acutely decrease activity in the ACC during resting state^33^ and during autobiographical memory recall^34^. If acute effects of psychedelic drugs on affect and the associated neurobiology are sustained after other acute effects of these drugs have resolved, these sustained effects may reveal a trans-diagnostic mechanism of the enduring therapeutic effects of psychedelics.

Available neuroimaging evidence may be interpreted to suggest that acute effects of psychedelic drugs on emotion perception (e.g.,^27^) are associated with alteration of bottom-up emotional reactivity through modulation of amygdala response to negative affective stimuli^28,31,32^. However, changes in emotion perception and positive and negative affect that are observed with psychedelic drugs could also reasonably result from changes in the top-down control of emotion that could lead to observed effects as down-stream results, and recent qualitative and self-report evidence supports this. A recent survey demonstrated that although psychedelics were not thought to reduce physiological components of craving and withdrawal, they may have reduced the affective components of craving and withdrawal^35^. Other reports identify increased “connection to life,” increased meaning-making^36^, and changes in other higher-level psychological factors^37^, as well as engagement with music^38^, as potential mechanisms underlying treatment efficacy.

Additional evidence abounds for a possible role of psychedelics in acutely decreasing resting-state connectivity within the default mode network (DMN)^33,39,40^, and between and within a number of sensory and cognitive brain networks^41,42^. Two reports have also provided evidence for a post-acute change in DMN connectivity after psilocybin administration, which was shown to be decreased two days after psilocybin in a cohort of long-term meditators^43^ and paradoxically increased one day after psilocybin in patients with treatment-resistant depression^44^. These findings argue for a potential neuroplastic effect of psilocybin on brain network function, loosely consistent with both *in vitro* and *in vivo* evidence for increased neuritogenesis and spinogenesis in cortical neurons in response to a wide range of classic, 5-HT_2A_ receptor agonist psychedelics^45^. Plasticity within higher-order cortical brain networks may allow for increased modulation of affect by top-down cognitive circuits.

The current open-label, within-subjects pilot study was conducted to examine whether a single administration of a high dose (25 mg/70 kg) of psilocybin could lead to an enduring increase in positive affect, enduring reduction in negative affect, enduring change in neural response to emotional stimuli, and enduring changes in resting state functional connectivity. A battery of self-report state and trait affect measures, including the Profile of Mood States (POMS)^46^, the State and Trait Anxiety Inventory (STAI)^47^, the Positive and Negative Affect Schedule – Form X (PANAS-X)^48^, the Depression, Anxiety, and Stress Scale (DASS)^49^, and the Dispositional Positive Emotion Scale (DPES)^50^, was completed one day before, one week after, and one month after administration of psilocybin, and responses were compared between time points to investigate the enduring effect of psilocybin on state and trait affect. Participants completed the Big Five Inventory (BFI)^51^ and the Tellegen Absorption Scale (TAS)^52^ one day before and one month after psilocybin, and responses were compared between time points to investigate the enduring effect of psilocybin on personality. One day before, one week after, and one month after psilocybin, participants also underwent fMRI measurements during rest and during the completion of three separate emotion processing tasks (the emotion discrimination task^15^, the emotion recognition task^53^, and an emotional conflict Stroop task^54^). fMRI data collected during emotional tasks were compared between time points to determine the enduring effects of psilocybin on response to emotional stimuli in the amygdala and ACC, and analyses were repeated with whole-brain data to determine if effects could be detected outside of a priori regions of interest (ROIs). Functional connectomes calculated from resting-state scans were compared between time points to determine the enduring effects of psilocybin on functional network connectivity.

## Results

### Psilocybin reduced negative affect and increased positive affect

Main effects of time point were observed on DASS stress (F[2,20] = 4.45, *p* = 0.025, *η*^2^_*p*_ = 0.284), PANAS negative affect (F[2,20] = 9.28, *p* = 0.0014, *η*^2^_*p*_ = 0.466), STAI state (F[2,20] = 3.91, *p* = 0.037, *η*^2^_*p*_ = 0.27) and trait (F[2,20] = 3.96, *p* = 0.036, *η*^2^_*p*_ = 0.277) anxiety, and POMS tension (F[2,20] = 6.37, *p* = 0.007, *η*^2^_*p*_ = 0.376), depression (F[2,20] = 5.46, *p* = 0.013, *η*^2^_*p*_ = 0.316), and total mood disturbance (F[2,20] = 5.66, *p* = 0.011, *η*^2^_*p*_ = 0.352) scale scores. Post-hoc tests demonstrated that DASS stress, PANAS negative affect, STAI state anxiety, and POMS tension, depression, and total mood disturbance scale scores were significantly lower 1 week after psilocybin compared to baseline and returned towards baseline ratings 1 month after psilocybin (Table 1). POMS depression was significantly greater at 1 month post-psilocybin compared to 1 week post-psilocybin. Ratings of trait anxiety were reduced 1-month post-psilocybin compared to baseline.

**Table 1:** Post-hoc tests of psilocybin effects on self-report measures of affect.

Measure	Baseline	1 week	Baseline vs 1 week	1 month	Baseline vs 1 month	1 week vs 1 month
	Mean (*SE*)	Mean (*SE*)	*z*, *p*	Mean (*SE*)	*z*, *p*	*z, p*
Joy (DPES)	5.45 (0.21)	**6.02 (0.17)**	**3.06, 0.007**	**5.92 (0.21)**	**2.92, 0.007**	0.08, 0.939
Content (DPES)	5.62 (0.24)	**6.27 (0.16)**	**2.97, 0.009**	**6.13 (0.23)**	**2.49, 0.026**	0.43, 0.667
Pride (DPES)	5.42 (0.22)	**5.96 (0.14)**	**2.85, 0.009**	**5.95 (0.18)**	**3.05, 0.007**	0.25, 0.805
Compassion (DPES)	5.75 (0.31)	**6.25 (0.26)**	**3.55, 0.001**	**6.00 (0.30)**	**3.18, 0.003**	0.26, 0.798
Amusement (DPES)	5.02 (0.31)	**5.60 (0.32)**	**3.11, 0.004**	**5.71 (0.31)**	**3.59, 0.001**	0.58, 0.565
Stress (DASS)	4.91 (1.06)	**2.00 (0.60)**	**2.95, 0.009**	3.27 (1.12)	1.81, 0.141	1.09, 0.276
Negative Affect (PANAS-X)	16.09 (1.00)	**12.36 (0.93)**	**4.28, 0.00006**	**13.91 (1.03)**	**2.50, 0.025**	1.68, 0.093
Tension (POMS)	4.82 (1.22)	**1.73 (0.49)**	**3.52, 0.0013**	3.82 (1.19)	1.23, 0.219	2.21, 0.054
Depression (POMS)	3.36 (0.81)	**0.55 (0.25)**	**3.20, 0.004**	2.64 (1.03)	0.87, 0.385	**2.29, 0.044**
Total Mood Disturbance (POMS)	3.64 (5.67)	**−10.5 (3.23)**	**3.37, 0.002**	−3.55 (6.19)	1.69, 0.181	1.59, 0.181
State Anxiety (STAI)	28.0 (2.41)	**22.82 (1.37)**	**2.80, 0.016**	25.64 (2.56)	1.31, 0.309	1.42, 0.309
Trait Anxiety (STAI)	31.36 (1.91)	28.55 (1.32)	2.11, 0.070	**27.55 (1.95)**	**2.65, 0.024**	0.60, 0.549

DASS: Depression, Anxiety, and Stress Scale; DPES: Dispositional Positive Emotion Scale; PANAS-X: Positive and Negative Affect Schedule - X; POMS: Profile of Mood States; STAI: State-Trait Anxiety Index; SE: standard error of the mean. Post-hoc tests were conducted using Tukey’s method for correcting for multiple comparisons^101^. Significant comparisons (α = 0.05, corrected) are indicated in bold font.

Main effects of time point were also observed on DPES joy (F[2,20] = 6.03, *p* = 0.009, *η*^2^_*p*_ = 0.36), content (F[2,20] = 5.11, *p* = 0.016, *η*^2^_*p*_ = 0.314), pride (F[2,20] = 5.85, *p* = 0.011, *η*^2^_*p*_ = 0.343), compassion (F[2,20] = 7.69, *p* = 0.004, *η*^2^_*p*_ = 0.44), and amusement (F[2,20] = 7.66, *p* = 0.004, *η*^2^_*p*_ = 0.435) scales. Post-hoc tests (Table 1) demonstrated that DPES scores were significantly greater both 1 week and 1 month after psilocybin compared to baseline. The only significant changes observed in personality between baseline and 1 month post-psilocybin (Table 1) were in conscientiousness (*t* = 2.33, *p* = 0.042, *d* = 0.738) and absorption (*t* = 3.55, *p* = 0.005, *d* = 1.122). Descriptive statistics for all self-report measures are presented in Supplementary Information (Table S1).

### Psilocybin led to changes in the neural response to affective stimuli

Response accuracy in the emotion recognition task was near ceiling for all emotional facial categories at all timepoints (mean accuracy 96.7%, SEM = 0.47%). No effect of condition (F[4,1421] = 1.171, p = 0.263), timepoint (F[2,1421] = 1.338, p = 0.1954), or interaction between timepoint and condition (F[8,1421] = 0.820, p = 0.5847) was observed on accuracy.

**Figure 1:**
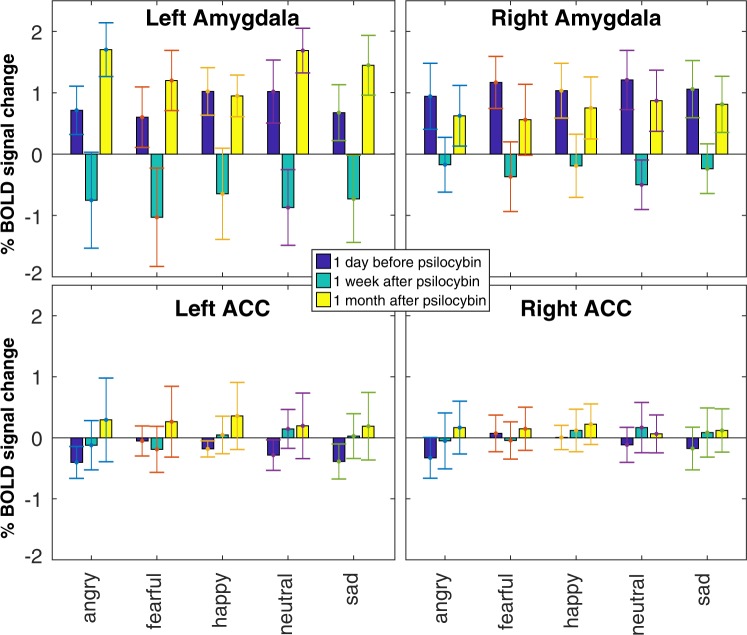
Longitudinal effects of a single high dose of psilocybin on amygdala and anterior cingulate response to facial emotional stimuli. Percentage of BOLD signal change (on the ordinate) for the [emotion > all stimuli] contrast are plotted for each emotional condition (angry, fearful, happy, neutral, and sad). Each panel of the figure presents contrast values for a different region of interest. Error bars are standard error. Dark blue bars plot values for baseline, turquoise bars plot values for 1 week post-psilocybin, and yellow bars for 1 month post-psilocybin. ACC: anterior cingulate cortex.

ROI analysis yielded a main effect of timepoint on BOLD response to stimuli in the emotion recognition task in left amygdala (*F*[2,165] = 6.38, *p* < 0.0005, *η*^2^_*p*_ = 0.098), right amygdala (*F*[2,165] = 7.54, *p* < 0.005, *η*^2^_*p*_ = 0.068), and left ACC (*F*[2,165] = 6.66, *p* < 0.05, *η*^2^_*p*_ = 0.053), but not right ACC (*F*[2,165] = 3.34, *p* = 0.108, *η*^2^_*p*_ = 0.026). No effect of emotional condition or interaction between timepoint and emotional condition in any ROI was observed. Post-hoc comparisons (Fig. 1) demonstrated a significant reduction in BOLD response to all facial stimuli in left amygdala (*t*[118] = 4.303, *p* < 0.00005, *d* = 0.79) and right amygdala (*t*[118] = 4.199, *p* = 0.00005, *d* = 0.77) at 1 week compared to baseline. Interestingly, both left (*t*[118] = 4.557, *p* < 0.00005, *d* = 0.83) and right (*t*[118] = 3.043, *p* < 0.005, *d* = 0.56) amygdala response to all stimuli returned to baseline levels at 1 month, compared to 1 week, with no significant difference in left amygdala (*t*[118] = 0.909, *p* = 0.365, *d* = 0.17) or right amygdala (*t*[118] = 0.841, *p* = 0.402, *d* = 0.15) at 1 month compared to baseline post-psilocybin. Individual data for amygdala response in the emotion recognition task are presented in Supplementary Information (Fig. S1). Exploratory associations between changes in self-report affect across time and changes in amygdala response across time in the emotion recognition task are also presented in Supplementary Information (Figs. S2–S4). No effects were observed in whole-brain voxel-wise analysis of the emotion recognition task.

Performance accuracy during the emotion discrimination task was near ceiling at baseline (92.04%, SEM = 1.7%) and increased from baseline to 1 week (94.7%, SEM = 1.4%, *t* = 3.089, *p* = 0.006) and 1 month (94.1%, SEM = 1.5%, *t* = 2.669, *p* = 0.014) after psilocybin. No differences were observed in amygdala or ACC response to the emotion discrimination task, between baseline, 1 week, or 1 month time points, and no significant effects were observed in whole-brain voxel-wise analyses of the emotion discrimination task.

### Psilocybin increased neural response to conflicting emotional information in decision-making circuits

Participants performed at ceiling across all timepoints in the emotional conflict Stroop task (98.9%, SEM = 0.21%). Classic Stroop interference was observed, with a main effect of task condition (emotionally congruent vs incongruent trials) on response accuracy (F[3,2575] = 5.704, p = 0.00069) and response time (F[3,2575] = 7.019, p = 0.00011) for the Stroop task, with fewer correct responses and greater response time for incongruent compared to congruent trials. No main effect of timepoint or interaction between timepoint and condition was observed in behavioral data.

**Figure 2:**
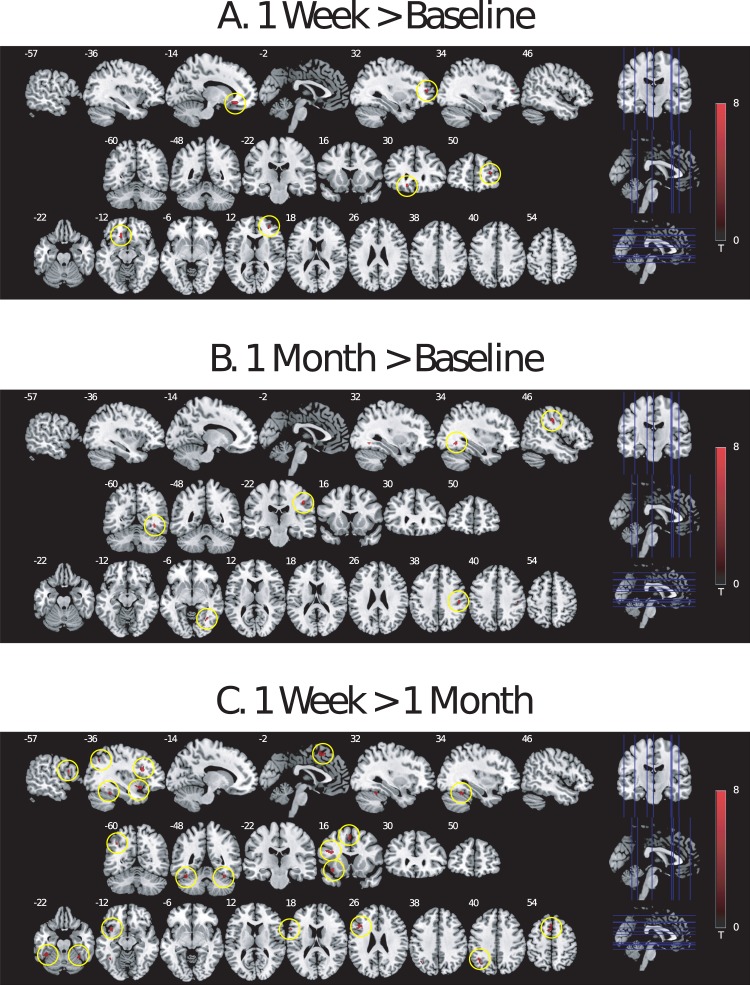
Longitudinal effects of a single high dose of psilocybin on brain response to high conflict trials in the emotional conflict Stroop task. T-values from whole-brain voxel-wise contrasts for high-demand incongruent (CI) greater than low-demand congruent (CC) trials [CI > CC] are presented for (**A**) 1 week post-psilocybin compared to baseline, (**B**) 1 month post-psilocybin compared to baseline, and (**C**) 1 week post-psilocybin compared to 1 month post-psilocybin. Each panel contains sagittal, coronal, and axial slices that display the significant clusters that were observed in the whole-brain general linear model analysis, with the in-plane coordinate for a given slice found in the top left-hand corner of each slice. Significant clusters in each slice are circled in yellow. CI: an incongruent Stroop trial that followed a congruent Stroop trial – this is a high-demand trial, as the trial involves both incongruent emotional information as well as a response-switch from responding to a congruent trial to responding to an incongruent trial; CC: a congruent Stroop trial that followed another congruent Stroop trial – this is a low-demand trial, as the trial involves congruent emotional stimuli and does not require response-switching from the previous trial.

No effect of task condition or time point on amygdala or ACC response was observed in ROI analyses for the emotional conflict Stroop task. However, whole-brain voxel-wise analysis of the emotional conflict Stroop task identified significant findings. It has been shown that trial-to-trial changes in task condition can alter cognitive control processes, with the greatest interference effects in Stroop-like paradigms being found in incongruent trials that follow congruent trials^19,55^. In a contrast of high-demand incongruent trials (incongruent trials that followed congruent trials, or CI trials) compared to low-demand congruent trials (congruent trials that followed congruent trials, or CC trials; [CI > CC] contrast), BOLD response increased from baseline to 1 week after psilocybin in dorsal lateral prefrontal (DLPFC) and medial orbitofrontal (MOFC) cortex (Fig. 2A; Table 2), and from baseline to 1 month after psilocybin in somatosensory cortex and fusiform gyrus (Fig. 2B; Table 2), but no decreases in BOLD responses (baseline >1 week, baseline >1 month) were observed. Greater BOLD response was also observed at 1 week compared to 1 month (1 week > 1 month) in a distributed network of primarily left hemisphere brain regions, including inferior frontal gyrus (IFG), anterior insula, parietal lobule, and fusiforum gyrus (Fig. 2C; Table 2), with no significant effects in the opposite contrast (1 month > 1 week).

**Table 2:** Longitudinal effects of a single high dose of psilocybin on brain response to high conflict trials in the emotional conflict Stroop task.

Contrast	Anatomical Region	x, y, z	k	T	Z
[CI > CC: 1 week] > [CI > CC: baseline]	L. dorsal lateral prefrontal cortex	32, 48, 12	22	10.88	4.78
	R. medial orbitofrontal cortex	−14, 30, −12	40	10.7	4.75
[CI > CC: 1 month] > [CI > CC: baseline]	R. postcentral gyrus	46, −22, 38	40	8.45	4.49
	R. fusiform gyrus	34, −60, −8	22	7.69	4.31
[CI > CC: 1 week] > [CI > CC: 1 month]	L. inferior frontal gyrus (pars triangularis)	−42, 20, 24	114	7.94	4.23
	L. anterior insula	−36, 14, −12	52	7.43	4.11
	L. inferior frontal gyrus (pars opercularis)	−56, 12, 22	23	8.23	4.29
	L. supplementary motor area	−6, 12, 56	82	6.46	3.85
	L. premotor area	−28, 6, 46	21	5.5	3.55
	L. inferior parietal lobule	−40, −54, 40	34	6.14	3.76
	L. fusiform gyrus	−40, −46, −24	67	5.79	3.65
	L. fusiform gyrus	−40, −54, −14	33	7.04	4.01
	R. fusiform gyrus	34, −50, −22	27	6.69	3.92

Significant clusters from whole-brain voxel-wise contrasts for high-demand incongruent (CI) greater than low-demand congruent (CC) trials [CI > CC], compared between 1 week and baseline ([CI > CC: 1 week] > [CI > CC: baseline]), and 1 week and 1 month ([CI > CC: 1 week] > [CI > CC: 1 month]) were thresholded at p(unc) < 0.0005, k ≥ 20. L: left; R: right; x, y, z: coordinates of the peak voxel in each cluster; k: cluster size. Anatomical regions for each cluster were determined using the SPM Anatomy Toolbox and the Duvernoy Brain Atlas. Whole-brain analyses were thresholded at p < 0.0005 (uncorrected), with cluster-forming threshold of p < 0.05 (uncorrected).

### Psilocybin increases resting state functional connectivity across brain networks

**Figure 3:**
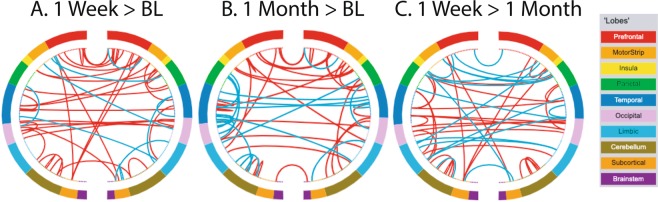
Longitudinal effects of a single high dose of psilocybin on the strength of static functional brain connectivity. Static functional connections (edges) that significantly increase (red lines) or decrease (blue lines) in strength (**A**) at 1 week compared to baseline, (**B**) at 1 month compared to baseline, and (**C)** at 1 week compared to 1 month are plotted in circle brains (https://bioimagesuiteweb.github.io/webapp/connviewer.html). The left and right side of each panel represents left and right hemispheres of the brain, respectively. Each dot in the inner ring of dots in each hemisphere corresponds to a node or region of interest within the brain, and the outer band of color provides a color code indicating the lobe of the brain within which each node resides. Color to lobe mapping is provided in the inset legend. Each edge is significantly different between time points (p < 0.05 after correction for multiple comparisons using the Bonferroni method).

**Figure 4:**
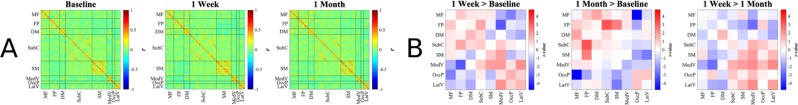
Effects of psilocybin on edge-wise and network-based static functional connectivity. (**A**) Static functional connectivity is shown for all pairwise functional connections (268 nodes × 268 nodes = 35,778 edges) at each time point. Each row and each column represents a single node (ROI) as defined by the Shen 268-node functional brain atlas^56^. The color of each off-diagonal cell in the connectome matrix represents the Pearson correlation value (r) for each edge (between the given nodes) in the brain. Nodes are grouped together in rows and columns by network as defined in the Shen atlas, with black lines marking the border between networks in the matrix. (**B**) Differences in static functional connectivity are shown within and between canonical networks for 1 Week > Baseline, 1 Month > Baseline, and 1 Week > 1 Month. Each row and column represents a single brain network as defined by the Shen 268-node functional brain atlas^56^. The diagonal cells represent differences in within-network connectivity between time points, and off-diagonal cells represent differences in between-network connectivity between time points. MF = medial frontal network, FP = frontoparietal network, DM = default mode network, SubC = subcortical-cerebellum network (including the salience network), SM = somatosensory-motor network, MedV = medial visual network, OccP = occipital pole network, and LatV = lateral visual network.

Out of 35,778 possible functional connections in the Shen atlas^56^, 695 were significantly different from zero (after Bonferroni correction) for at least one time point. Functional connectivity increased across the brain from baseline to 1 week after psilocybin (greater connectivity strength for 38 edges and less for 10 edges) and this pattern persisted at one month (greater connectivity strength for 29 edges and less for 18 edges at 1 month; Fig. 3). Of the 29 edges that showed greater connectivity at 1 month post-psilocybin, 7 of these were the same edges as those that increased from baseline to 1 week post-psilocybin, and these edges were evenly distributed across different brain lobes and networks. Changes in static functional connectivity did not follow any discernable network pattern (Fig. 4A), however there were more numerical increases than numerical decreases in functional connectivity within and between networks one week and one month after psilocybin compared to baseline (Fig. 4B).

Measures of dispersion of connectivity strengths within and between networks were unaffected across time points (Figs. S5–S8).

## Discussion

The current open-label pilot study identified four key sustained effects of a single high dose of psilocybin on affect and the neural correlates of affective processing. First, negative affect was decreased 1 week post-psilocybin and returned to baseline levels at 1 month post-psilocybin. Second, there were decreases in amygdala responses to emotional stimuli 1 week post-psilocybin that rebounded at 1 month post-psilocybin. Third, there were increased responses in reward-learning, attention, and decision-making circuits 1 week post-psilocybin, and increased responses in somatosensory and fusiform gyrus 1 month post-psilocybin, during high-demand incongruent trials in the emotional conflict Stroop task. Finally, there were global increases in functional connectivity at both 1 week and 1 month post-psilocybin.

A notable feature of the current report is that the reported effects of psilocybin were observed well after psilocybin would have been eliminated from the body and beyond expected transient effects of receptor trafficking that may be occurring after psilocybin administration. The half-life of psilocybin and psilocin (the active metabolite of psilocybin) is roughly 3 hours^57,58^, indicating that over 50 half-lives of the drug had passed before the 1 week time point, ensuring elimination of the drug from each participant. Further, while the 5-HT_2A_ receptor is known to internalize rapidly with both agonism and antagonism, it is thought to be re-expressed roughly 24–48 hours after internalization (in the absence of chronic engagement)^59^, and thus any transient changes in receptor dynamics related to psilocybin administration would be resolved by the 1 week time point. Rather than receptor trafficking or other residual pharmacological effects, the reported findings might better be explained by a neuroplastic period during which the neural processing of affective stimuli is altered.

The sustained decreases in negative affective states and traits, increases in positive affective states and traits, and decreases in amygdala responses to emotional stimuli that were observed in this trial all resemble reported acute effects of psilocybin^27,28^. The observed changes in MOFC, DLPFC, IFG, insula, parietal, and fusiform response to conflicting trials, however, are unexpected findings that may reveal a potential top-down mechanism underlying the sustained effects of psilocybin on affect and brain function.

### Psilocybin may increase the top-down control of emotional processes

The DLPFC is broadly implicated in a number of tasks spanning the domains of working memory^60^, decision making^61^, and emotion regulation^62^. Hypoactive DLPFC response to emotional interference has been demonstrated in major depressive disorder, suggesting a deficit in the neural circuitry underlying emotion regulation and top-down control of emotionally conflicting information^63^, and this hypoactive response has been shown to recover with antidepressant treatment^64^. DLPFC has also been shown to exert top-down influence on amygdala response during emotion regulation^62^. Reduced DLPFC recruitment and enhanced amygdala recruitment during down-regulation of negative emotion have been identified across a range of disorders including mood and substance use disorders^65^.

MOFC response is observed in a wide range of decision-making tasks^66,67^, and may code the reward value of reinforcers, with an anterior-to-posterior gradient within the OFC suggesting that more abstract reinforcers elicit more anterior OFC response^68^. The observed increase in anterior MOFC at 1 week post-psilocybin is consistent with increased sensitivity to the abstract reinforcer of positive emotional stimuli. The amygdala and MOFC also have dense bidirectional structural connections that are understood to facilitate top-down modulation of salience detection and reward learning^68^.

Taken together, increased DLPFC and MOFC response to high-conflict trials in the emotional conflict Stroop at 1 week post-psilocybin task may reflect greater top-down control of emotionally conflicting information and suppression of amygdala response to negative affective stimuli, which may lead to a shift in the relative salience of positive and negative affective information in the environment, and an overall shift in affect. A lack of observed change in behavior during the emotional conflict Stroop may indicate that behavioral performance was already at ceiling during baseline. However, this leaves open the possibility that executive control over emotionally conflicting information was less efficient at 1 week post-psilocybin, leading to greater recruitment of DLPFC and MOFC to maintain the same level of behavior.

IFG has been implicated in supporting a domain-general interference resolution process^69^. Activity in the anterior insula is understood to contribute to interoceptive mapping^70^, and both IFG and insula may be involved in the appraisal of social emotional stimuli^71^. The fusiform gyrus contains a number of functionally-defined sub-regions dedicated to stimulus-specific object recognition^72–74^, with specialized regions that respond to facial stimuli^75,76^. Greater recruitment of these brain regions in response to conflicting stimuli at 1 week compared to 1 month post-psilocybin may reflect increased attentional load and more acute visceral representation of emotionally conflicting information.

### Comparison of current findings with recently reported literature

Publications from only three other studies have reported effects of a psychedelic drug that lasted beyond the acute drug effect. One such study showed increased amygdala response to negative facial affect stimuli as measured using fMRI one day after psilocybin administration in a cohort of patients with treatment-resistant depression^77^. That finding is somewhat puzzling because amygdala response to negative stimuli is abnormally increased in patients with depression^18,78^, and one would expect an antidepressant response to be accompanied by normalization (e.g., decrease) in amygdala response to negative stimuli^79,80^. A potential explanation for this short-term rebound effect in amygdala response may be that increased amygdala response one day after psilocybin administration reflects a transient alteration of serotonergic signaling, as 5-HT_2A_ receptors are well-known to readily internalize with both agonism and antagonism^59^, including after administration of classic psychedelics^81^, with re-expression occurring up to 48 hours later^59^. It is important to note, however, that increased amygdala reactivity one day after psilocybin was associated with therapeutic outcomes in this sample, and it may be that a subacute rebound process one day after psilocybin is followed by a subsequent drop in amygdala responsiveness at one week (as in the current sample). It is also important to note that any apparent differences between this and previous studies in the direction of findings regarding amygdala response may be a function of the different populations studied, where the current study is investigating effects in healthy volunteers and the previous study was conducted in patients with treatment resistant depression.

While no one brain network stood out as uniquely (or significantly) altered with respect to post-psilocybin connectivity in the current report, the pattern of increased connectivity across brain networks that was sustained 1 week and 1 month post-psilocybin is generally consistent with reported acute effects of psilocybin^41^, where connectivity between and among a number of canonical brain networks is increased. The current findings are also consistent with recent reports that demonstrated increased within-network connectivity in the DMN the day after the second of two psilocybin administrations in patients with treatment resistant depression^44^, two days after a single dose of psilocybin was administered to individuals in a 5-day meditation retreat^43^, and the day after administration of a closely related substance, ayahuasca, to healthy participants^82^. It should be noted, though, that the aforementioned reports of connectivity changes in the DMN one or two days after psychedelic drug administration were restricted to a priori analysis of the DMN, and these studies did not report on the between- or within-network connectivity of other canonical networks. In addition, these studies differed from the current report in that all previous studies conducted resting-state imaging with eyes closed, whereas the current study conducted resting-state imaging with eyes open. Also, while the current study collected 16 minutes worth of resting-state data, previous studies measured 7 or 8 minute resting-state scans, which may yield less reliable resting-state connectivity estimates than scans of 12 minutes or longer^83^.

Changes in personality traits after psilocybin administration are also notable. While previous studies have noted trait absorption to be a predictor of response to psychedelic drugs^84,85^, this is the first demonstration that psilocybin administration may lead to a shift in absorption. Numerical increases in openness and extroversion and numerical decreases in neuroticism are consistent with previous effects of psilocybin on Big-Five personality traits^86,87^, but surprisingly, the strongest effect of psilocybin on Big-Five traits was on an increase in conscientiousness (*d* = 0.738). Whether these effects are idiosyncratic to the given sample, generalize to other healthy participant samples, or have relevance to therapeutic outcomes in patient populations has yet to be determined.

### Limitations

All conclusions in the current report are limited by small sample size. However, concerns regarding sample size may be mitigated by the moderate to strong effect sizes that were observed across both self-report and neurobiological outcomes. Lack of a placebo or positive control comparison, the open-label nature of the study, and lack of multiple time points prior to psilocybin administration leaves open the possibility that some of the reported effects are due to expectancy, experimental demand characteristics, and learning or habituation effects. These concerns are somewhat mitigated by the fact that negative affect and task-based fMRI effects at 1 week return towards baseline levels at 1 month post-psilocybin. Nonetheless, replication of this study in a larger sample with compelling control conditions is warranted.

## Conclusions

The current report provides preliminary evidence that psilocybin administration may lead to shifts in affect and the neural correlates of affective processing that endure beyond acute drug effects. Within a dimensional or domain-based taxonomy of brain function and pathology, the reported findings are consistent with a trans-diagnostic process that may underlie both mood and substance use disorders. Reduction of negative affect may undermine ruminative processes that contribute to the development and maintenance of mood disorders, and these effects are consistent with psychological and neural changes that might be expected to accompany antidepressant effects of psilocybin. Disruption of the negative components of craving and withdrawal may undermine the development and maintenance of substance use disorders, consistent with psychological and clinical changes observed in patients with tobacco and alcohol use disorders. Reported findings may also account for long-term positive changes in mood, attitude, and well-being that have been reported in healthy individuals^88,89^.

While both negative affect and brain response to affective stimuli were reduced 1 week after psilocybin, they rebounded at the 1 month timepoint, suggesting that psilocybin may have initiated a dynamic and neuroplastic process that was sustained for at least a number of weeks. It is possible that such a neuroplastic period may allow for a more enduring shift towards positive affective. The observed increase in functional connectivity strength indiscriminately across networks may reflect a domain-general cortical plasticity process supporting the observed changes in affective processing, consistent with preclinical evidence for psychoplastogenic properties of psychedelic drugs^45,90^. Overall, the current findings identify negative affect as a potential therapeutic target of psilocybin.

## Methods

### Participants

Twelve volunteers (7 females, mean age 32.1 ± 7.5 years) took part in this open-label, within-subjects, longitudinal pilot study. Volunteers were included if they were right-handed, between the ages of 18–45, medically healthy (as determined by medical history, physical examination, an electrocardiogram, blood analysis, and urine testing for common drugs of abuse), and psychiatrically healthy (as determined by the Structured Clinical Interview for DSM-IV). Individuals were excluded for MRI contraindications (including past head trauma, claustrophobia, presence of certain implants, and/or non-removable ferrous metals) as well as potential psilocybin contraindications (personal or family histories of psychotic or bipolar disorder, history within past 5 years of moderate or severe substance use disorder, and taking medications with a psychoactive or CNS effect). A urine pregnancy test (for females) and a urine test for common drugs of abuse (for all participants) was required to be negative during screening and the morning of drug administration.

The sample was racially homogenous (100% Caucasian), more than half (58.3%) were married at the time of their participation, 83.3% had earned a Bachelor’s degree or higher, and all reported limited lifetime use of hallucinogens (median of 1, range 1–4 uses), with the most recent use occurring an average of 8.3 years ago. This study was registered at ClinicalTrials.gov (NCT02971605, registered on November 23, 2016). All participants provided informed consent in accordance with the Common Rule and the Declaration of Helsinki. All procedures were approved by the Johns Hopkins University School of Medicine Institutional Review Board, and participants were compensated a total of $240 upon completion of the study.

### Study procedures

Upon enrollment, participants underwent preparation, acute care, and aftercare for psilocybin administration sessions following published safety guidelines^91^. Participants were assigned two session monitors with whom they met during two preparatory meetings before drug administration, for a total of roughly eight hours of preparation time. During preparatory meetings, participants recounted life history and important lifetime events, received training on and practiced each of three emotion tasks that would be performed during MRI assessments (*see “Affective Tasks” below*), and monitors instructed participants on the range of possible experiences that may be encountered during acute drug effects. Emotion task practice sessions were included to ensure that participants were familiar with all tasks before MRI procedures commenced, and to minimize initial learning effects on these tasks. Participants then completed a single psilocybin administration session lasting roughly 7 hours and using established procedures^91^ based on several previous and ongoing studies with healthy participants^89,92–95^ and clinical populations^1,4^. Participants returned the day after their psilocybin session to meet with study staff and review the previous day’s psilocybin session.

#### Psilocybin session

Participants consumed a small low-fat breakfast >1-hour prior to arriving at the Behavioral Pharmacology Research Unit at the Johns Hopkins Bayview Medical Center. Participants remained recumbent on a couch under the supportive supervision of two study staff after ingesting a capsule containing a high dose of psilocybin (25 mg/70 kg) that was prepared by our research pharmacy. Blood pressure, heart rate, and staff ratings of participant behavior were assessed as safety measures at 0, 30, 60, 120, 180, 240, 300, and 360 minutes after capsule administration.

#### Questionnaires

A battery of questionnaires was completed one day before, one week after, and one month after psilocybin administration to assess emotional function. The Positive and Negative Affect Scale - X (PANAS-X)^48^ is a 60-item adjective rating scale with a 5-point response format (0 – very slightly or not at all, 1 – a little, 2 – moderately, 3 – quite a bit, 4 - extremely) that is scored into general positive and negative affect sub-scales, as well as a number of facets of positive and negative affect. Participants were asked to indicate the degree to which they generally feel (“that is, how you feel on the average”) the different feelings and emotions described by each adjective. The Profile of Mood States (POMS)^46^ is a 65-item rating scale with a 5-point response format (0 – Not at all, 1 – a little, 2 – moderately, 3 – quite a bit, 4 - extremely) that is scored into seven sub-scales (tension, depression, anger, fatigue, confusion, vigor, and total mood disturbance). Participants were asked to indicate the degree to which each item described how they had been feeling during the past week including today. The Dispositional Positive Emotions Scale (DPES)^50^ is a 38-item Likert scale with a 7-point response format (with response anchors at 1 “Strongly disagree”, 4 “Neither agree nor disagree”, and 7 “Strongly agree”) that is scored into seven sub-scales (joy, content, pride, love, compassion, amusement, and awe). Participants were asked to think about each statement and decide how much they agree or disagree with it. The Depression Anxiety Stress Scale (DASS)^49^ is a 21-item rating scale with a 4-point response format (0 – did not apply to me at all, 1 – applied to me to some degree, or some of the time, 2 – applied to me to considerable degree, or a good part of the time, 3 – applied to me very much, or most of the time) that is scored into three sub-scales (depression, anxiety, and stress). Participants were asked to indicate how much each statement in the DASS applied to them over the past week. The State-Trait Anxiety Inventory (STAI)^47^ is a 40-item rating scale with a 4-point response format (0 – almost never, 1 – sometimes, 2 – often, 3 – almost always) that is scored into two sub-scales (state anxiety and trait anxiety). For “state” anxiety questions, participants were asked to select the response for each item that best describes how they feel “right now, that is, at this moment”. For “trait” anxiety questions, participants were asked to select the response that best describes how they “generally feel, that is, most of the time”.

Participants also completed measures of personality at screening and again one month after psilocybin. The Big Five Inventory (BFI)^51^ is a 44-item Likert scale with a 5-point response format (1 – Disagree strongly, 2 – Disagree a little, 3 – Neither agree nor disagree, 4 – Agree a little, 5 – Agree strongly) that is scored into five sub-scales (extraversion, neuroticism, agreeableness, conscientiousness, openness). The Tellegen Absorption Scale (TAS)^52^ is a 34-item rating scale with a 4-point response format (with response anchors at 0 “Never” and 3 “Always”) that is scored into a single total score for absorption.

#### MRI Assessments

One day before, one week after, and one month after psilocybin administration, participants completed the emotion discrimination, emotion recognition, and emotional conflict Stroop tasks in that order, with an 8-minute eyes-open resting-state scan between each pair of tasks (16 total minutes of resting scans for each visit), during the measurement of blood-oxygenation level-dependent (BOLD) signal using echo-planar imaging (EPI; TR/TE = 2200/30 ms, flip angle = 75°, voxel size = 3 mm^3^, 37 axial slices collected in an interleaved fashion with a 1 mm slice gap, with SENSE acceleration factor = 2). All scanning procedures were performed on a Philips 3T MRI scanner equipped with a 32-channel head coil at the F.M. Kirby Research Center for Functional Brain Imaging at the Kennedy Krieger Institute in Baltimore, MD. Each scanning session lasted 60 minutes.

Task performance during MRI sessions began with a short practice task in the scanner before MRI measurement, followed by the full task performance during MRI measurement. All facial emotional stimuli were selected from the NimStim Emotional Facial Expression database^96^, and balanced within task and between conditions to the degree possible based on sex, race, and the frequency of mouth opened vs closed in each stimulus. Visual stimuli were projected onto a frosted Plexiglas shield at the open end of the scanner bore, which was viewed through a mirror placed on the head coil. Participants made responses using a fiber-optic, MR-safe response device. Stimuli and responses were presented and recorded using Presentation Software (Neurobehavioral Systems, Inc. Berkeley, CA).

Emotion discrimination.  During this task, participants viewed an array of three images (one at the top of the screen and two at the bottom of the screen) containing either three emotional (fearful or angry) facial expressions or three geometric shapes (vertically or horizontally oriented ellipsoids)^16,17,28^. Participants were instructed to press a button (either in the right or left hand) to indicate the image on the bottom of the screen (either the right or the left image) that matched the image at the top of the screen. Participants completed four 30 s blocks of face-matching trials interleaved between five 30 s blocks of shape-matching trials. Each block began with a 3 s cue (“match faces” or “match shapes”) followed by 6 trials (4.5 s per trial) and a 500 ms inter-stimulus interval (total task time: 4 m 57 s).

Emotion recognition. In this task, participants are presented with a series of happy, sad, fearful, angry, and neutral facial expressions and are instructed to press a button to identify the emotion expressed on each face^53,97,98^. Sixty stimuli (12 stimuli for each emotion) are presented one at a time for 4 seconds each, with a jittered ISI averaging 3 s, and 15 s of rest at the beginning and end of the task, for a total task time of 7 minutes and 30 seconds. An equal number of male and female faces were presented for each emotion. The order of emotions was pseudorandomized according to a genetic algorithm to maximize the statistical separation of each condition^99^, but within each emotion condition, the order of actual stimuli is randomized.

Emotional conflict Stroop. This task requires that participants identify the valence of emotional facial expressions (targets) with overlaid emotional words (distractors)^22,54^. Emotional facial expressions consist of 18 happy and 18 sad emotional faces (9 male and 9 female each), matched between emotional conditions on strength of emotional valence, and presented in pseudorandom order. Emotional words consisted of 18 positively valenced and 18 negatively valenced emotional words from the Affective Norms for English Words (ANEW)^100^ that are matched between valence conditions on the intensity of valence (degree of pleasure vs displeasure), arousal, dominance, and word length (in characters), and paired in pseudorandom order with a facial emotional stimuli. A given target and distractor pair may have congruent or incongruent emotional valence. Stimuli are pseudorandomized to control for the order of congruent (C) and incongruent (I) stimuli, balancing for order effects for the following sequences across gender and emotional valence of the target stimulus: congruent trials that follow a previous contgruent trial (CC), congruent trials that follow a previously incongruent trial (IC), incongruent trials that follow a previous incongruent trial (II), and incongruent trials that follow a previous congruent trial (CI).

### Analysis

#### Self-report questionnaire analysis

Mixed-effects, repeated measures one-way ANOVAs were used to determine the persisting effects of psilocybin on self-report affect measures, comparing each measure between each time point (baseline, 1-week, and 1-month post-psilocybin). Where a significant main effect was observed, we then followed up with post-hoc comparisons between each time point, corrected for multiple comparisons using Tukey’s method for multiple comparisons of all pairwise means^101^. Paired *t*-tests were used to test for changes personality measures between screening and 1-month post-psilocybin.

#### Preprocessing and analysis of task-based BOLD data

All task-based BOLD data underwent preprocessing, region of interest (ROI) extraction, and ROI analysis to determine the response of the left and right amygdala and left and right ACC to task conditions in each fMRI task. Preprocessing steps consisted of slice timing correction, realignment/motion correction, normalization to an EPI template registered to MNI space^102^, and smoothing using a 6 mm FWHM kernel. The first eigenvariable of all voxels within four ROIs (left and right amygdala and left and right ACC) was extracted for each subject and each scan and submitted to separate subject-level general linear model (GLM) analyses for each affective task at each time point (baseline, 1 week post-psilocybin, and 1 month post-psilocybin).

Subject-level GLM design matrices consisted of six motion parameters from realignment, a motion sensoring or “scrubbing” regressor generated using outlier detection and intermediate settings (global-signal z-value threshold = 5, subject-motion mm threshold = 0.9) in the ART toolbox^103^, the mean signal within each run, a linear term to model signal drift, a regressor to model all button-presses made by the participant, and regressors of interest for each task. The design matrix for the emotion discrimination task included regressors of interest for face blocks and shape blocks, and a [face > shapes] contrast was fit as the contrast of interest for each subject and time point. The design matrix for the emotion recognition task included a regressor indicating the onset of every stimulus, and separate regressors of interest for each emotional face condition (happy, angry, sad, fearful, and neutral). An emotion greater than all stimulus contrast was fit for each emotional condition ([happy > all stimuli], [angry > all stimuli], etc). The design matrix for the emotional conflict Stroop task included regressors of interest for each of the four first-order sequence types (congruent trials that follow a congruent trial, or CC, incongruent trials that follow a congruent trial, or CI, incongruent trials that follow an incongruent trial, or II, and congruent trials that follow an incongruent trial, or IC). Two contrasts of interest were fit: one for all incongruent greater than all congruent trials ([CI & II > CC & IC]), and one for high-demand incongruent greater than low-demand congruent trials ([CI < CC]).

SPM12 (http://www.fil.ion.ucl.ac.uk/spm/software/spm12/) was used to preprocess all data, and SPM12, MaRSBaR (http://marsbar.sourceforge.net), and MATLAB (R2017a, version 9.2.0.556344) were used to conduct GLM analyses. A one-way ANOVA was fit to subject-level ROI contrasts to determine a main effect of time-point on BOLD response in each ROI for each task. Post-hoc comparisons were conducted using *t*-tests, corrected for multiple comparisons using the Holm-Bonferroni method^104^. Analyses were repeated as exploratory whole-brain voxel-wise general linear models to investigate potential effects outside of hypothesized areas. Whole-brain analyses were thresholded at p < 0.0005 (uncorrected), with cluster-forming threshold of p < 0.05 (uncorrected).

#### Resting state fMRI analysis

Resting-state data were preprocessed as task-based data and then submitted to simultaneous^105,106^ bandpass filtering (0.009–0.08 Hz) and regression of nuisance parameters. Nuisance parameters consisted of linear trends, the first 5 eigenvectors of cerebrospinal fluid and white matter signal (identified using masks derived from segmented and normalized T1-weighted structural images), 6 motion parameters from realignment, and a motion sensoring or “scrubbing” regressor generated using outlier detection and intermediate settings (global-signal z-value threshold = 5, subject-motion mm threshold = 0.9) in the ART toolbox^103^. Preprocessed and nuisance-regressed data were then parcellated using the Shen 268-node functional brain atlas^56^. Voxels within each node were averaged at each acquisition to produce 268 time series (one for each node) for each participant. One subject was excluded from resting-state analysis for missing resting-state data from the 1 week time-point.

Static functional connectivity between each edge (each pair-wise set of nodes from the Shen atlas) was calculated using Pearson correlations. These values and all other correlations were Fisher z-transformed for all statistics. To explore differences in whole brain static connectivity, significant edges (negative and positive) were identified using separate one-sample *t* tests across participants for each edge and timepoint, thresholded using Bonferroni correction for all 35,778 edges. Although statistically conservative, this procedure yields the most reliable edges across our relatively small sample. All edges that survived this thresholding for at least one time point were then contrasted between time points (baseline vs. one week and baseline vs. one month) using paired *t* tests (α = 0.05).

Two resting-state scans were collected at each MRI visit, and all resting-state dependent variables were averaged within-subject at each time point and each edge before analysis. Nodes of the Shen atlas cluster into eight canonical functional networks: medial frontal, frontoparietal, default mode, subcortical-cerebellum (including salience), motor, visual I (medial), visual II (occipital pole), and visual association (lateral), yielding 8 additional within-network observations and 28 between-network observations for each outcome measure (static functional connectivity, DCC, and entropy). In order to explore within and between network differences, all edges within each network, or all edges between each pair of networks were averaged and compared across time points (via *t* test). Visual analysis of the matrix of *t*-values was used to identify obvious patterns in connectivity change, but should be interpreted with caution.

## Supplementary information

Supplementary Information.

## Data Availability

Data can be made available to qualified investigators upon reasonable request.

## References

[1] Griffiths RR (2016). Psilocybin produces substantial and sustained decreases in depression and anxiety in patients with life-threatening cancer: A randomized double-blind trial. J. Psychopharmacol..

[2] Ross S (2016). Rapid and sustained symptom reduction following psilocybin treatment for anxiety and depression in patients with life-threatening cancer: a randomized controlled trial. J. Psychopharmacol..

[3] Carhart-Harris RL (2016). Psilocybin with psychological support for treatment-resistant depression: an open-label feasibility study. Lancet Psychiatry.

[4] Johnson MW, Garcia-Romeu A, Cosimano MP, Griffiths RR (2014). Pilot study of the 5-HT2AR agonist psilocybin in the treatment of tobacco addiction. J. Psychopharmacol..

[5] Garcia-Romeu A, Griffiths RR, Johnson MW (2014). Psilocybin-occasioned mystical experiences in the treatment of tobacco addiction. Curr. Drug. Abuse Rev..

[6] Bogenschutz MP (2015). Psilocybin-assisted treatment for alcohol dependence: a proof-of-concept study. J. Psychopharmacol..

[7] Bogenschutz MP (2018). Clinical Interpretations of Patient Experience in a Trial of Psilocybin-Assisted Psychotherapy for Alcohol Use Disorder. Front. Pharmacol..

[8] Johnson MW, Garcia-Romeu A, Griffiths RR (2017). Long-term follow-up of psilocybin-facilitated smoking cessation. Am. J. Drug. Alcohol. Abuse.

[9] Hofmann SG, Sawyer AT, Fang A, Asnaani A (2012). Emotion dysregulation model of mood and anxiety disorders. Depress. Anxiety.

[10] Boumparis N, Karyotaki E, Kleiboer A, Hofmann SG, Cuijpers P (2016). The effect of psychotherapeutic interventions on positive and negative affect in depression: A systematic review and meta-analysis. J. Affect. Disord..

[11] Bourke C, Douglas K, Porter R (2010). Processing of facial emotion expression in major depression: a review. Australian N. ZealJ. Psychiatry.

[12] Volkow ND, Koob GF, McLellan AT (2016). Neurobiologic Advances from the Brain Disease Model of Addiction. N. Engl. J. Med..

[13] LeDoux, J. *The Emotional Brain: The Mysterious Underpinnings of Emotional**Life*. (Touchstone, 1998).

[14] LeDoux J (2007). The amygdala. Curr. Biol..

[15] Hariri AR, Bookheimer SY, Mazziotta JC (2000). Modulating emotional responses: effects of a neocortical network on the limbic system. Neuroreport.

[16] Hariri AR, Tessitore A, Mattay VS, Fera F, Weinberger DR (2002). The amygdala response to emotional stimuli: a comparison of faces and scenes. Neuroimage.

[17] Hariri AR, Mattay VS, Tessitore A, Fera F, Weinberger DR (2003). Neocortical modulation of the amygdala response to fearful stimuli. Biol. Psychiatry.

[18] Almeida JRC, Versace A, Hassel S, Kupfer DJ, Phillips ML (2010). Elevated amygdala activity to sad facial expressions: a state marker of bipolar but not unipolar depression. Biol. Psychiatry.

[19] Botvinick MM, Braver TS, Barch DM, Carter CS, Cohen JD (2001). Conflict monitoring and cognitive control. Psychol. Rev..

[20] Botvinick MM, Cohen JD, Carter CS (2004). Conflict monitoring and anterior cingulate cortex: an update. Trends Cogn. Sci..

[21] van Veen V, Cohen JD, Botvinick MM, Stenger VA, Carter CS (2001). Anterior cingulate cortex, conflict monitoring, and levels of processing. Neuroimage.

[22] Etkin A, Egner T, Kalisch R (2011). Emotional processing in anterior cingulate and medial prefrontal cortex. Trends Cogn. Sci..

[23] Wager TD (2013). An fMRI-based neurologic signature of physical pain. N. Engl. J. Med..

[24] Kross E, Berman MG, Mischel W, Smith EE, Wager TD (2011). Social rejection shares somatosensory representations with physical pain. Proc. Natl. Acad. Sci. USA.

[25] Drevets WC, Savitz J, Trimble M (2008). The subgenual anterior cingulate cortex in mood disorders. CNS Spectr..

[26] Rocha JM (2019). Serotonergic hallucinogens and recognition of facial emotion expressions: a systematic review of the literature. Ther. Adv. Psychopharmacol..

[27] Kometer M (2012). Psilocybin biases facial recognition, goal-directed behavior, and mood state toward positive relative to negative emotions through different serotonergic subreceptors. Biol. Psychiatry.

[28] Kraehenmann R (2015). Psilocybin-Induced Decrease in Amygdala Reactivity Correlates with Enhanced Positive Mood in Healthy Volunteers. Biol. Psychiatry.

[29] Schmidt A, Kometer M, Bachmann R, Seifritz E, Vollenweider F (2013). The NMDA antagonist ketamine and the 5-HT agonist psilocybin produce dissociable effects on structural encoding of emotional face expressions. Psychopharmacol..

[30] Dolder PC, Schmid Y, Müller F, Borgwardt S, Liechti ME (2016). LSD Acutely Impairs Fear Recognition and Enhances Emotional Empathy and Sociality. Neuropsychopharmacology.

[31] Kraehenmann R (2015). The mixed serotonin receptor agonist psilocybin reduces threat-induced modulation of amygdala connectivity. Neuroimage Clin..

[32] Grimm O, Kraehenmann R, Preller KH, Seifritz E, Vollenweider FX (2018). Psilocybin modulates functional connectivity of the amygdala during emotional face discrimination. Eur. Neuropsychopharmacol..

[33] Carhart-Harris RL (2012). Neural correlates of the psychedelic state as determined by fMRI studies with psilocybin. Proc. Natl. Acad. Sci. USA.

[34] Carhart-Harris RL (2012). Implications for psychedelic-assisted psychotherapy: functional magnetic resonance imaging study with psilocybin. Br. J. Psychiatry.

[35] Johnson MW, Garcia-Romeu A, Johnson PS, Griffiths RR (2017). An online survey of tobacco smoking cessation associated with naturalistic psychedelic use. J. Psychopharmacol..

[36] Preller KH (2017). The Fabric of Meaning and Subjective Effects in LSD-Induced States Depend on Serotonin 2A Receptor Activation. Curr. Biol..

[37] Roseman L, Nutt DJ, Carhart-Harris RL (2017). Quality of Acute Psychedelic Experience Predicts Therapeutic Efficacy of Psilocybin for Treatment-Resistant Depression. Front. Pharmacol..

[38] Kaelen M (2018). The hidden therapist: evidence for a central role of music in psychedelic therapy. Psychopharmacol..

[39] Carhart-Harris RL (2014). The entropic brain: a theory of conscious states informed by neuroimaging research with psychedelic drugs. Front. Hum. Neurosci..

[40] Palhano-Fontes F (2015). The psychedelic state induced by ayahuasca modulates the activity and connectivity of the default mode network. PLoS ONE.

[41] Roseman L, Leech R, Feilding A, Nutt DJ, Carhart-Harris RL (2014). The effects of psilocybin and MDMA on between-network resting state functional connectivity in healthy volunteers. Front. Hum. Neurosci..

[42] Carhart-Harris RL (2016). Neural correlates of the LSD experience revealed by multimodal neuroimaging. Proc. Natl. Acad. Sci. USA.

[43] Smigielski L, Scheidegger M, Kometer M, Vollenweider FX (2019). Psilocybin-assisted mindfulness training modulates self-consciousness and brain default mode network connectivity with lasting effects. Neuroimage.

[44] Carhart-Harris RL (2017). Psilocybin for treatment-resistant depression: fMRI-measured brain mechanisms. Sci. Rep..

[45] Ly C (2018). Psychedelics Promote Structural and Functional Neural Plasticity. Cell Rep..

[46] McNair, D M., Lorr, Maurice & Droppleman, Leo F. *Profile of Mood States (POMS)*. (Multi-Health Systems Inc, 1971).

[47] Spielberger, C. D. Manual for the State-Trait Anxiety Inventory STAI (Form Y) (‘Self-Evaluation Questionnaire’) (1983).

[48] Watson, D & Clark, L A. *The PANAS-X: Manual for the Positive and Negative Affect Schedule - Expanded Form*. (1994).

[49] Henry JD, Crawford JR (2005). The short-form version of the Depression Anxiety Stress Scales (DASS-21): construct validity and normative data in a large non-clinical sample. Br. J. Clin. Psychol..

[50] Shiota MN, Keltner D, John OP (2006). Positive emotion dispositions differentially associated with Big Five personality and attachment style. J. Posit. Psychol..

[51] John, O. P., Naumann, L. P. & Soto, C. J. Paradigm Shift in the Integrative Big-Five Trait Taxonomy: History, Measurement, and Conceptual Issues. In *Handbook of personality: Theory and research* 114–158 (Guilford Press, 2008).

[52] Tellegen A, Atkinson G (1974). Openness to absorbing and self-altering experiences (‘absorption’), a trait related to hypnotic susceptibility. J. Abnorm. Psychol..

[53] Gur RC (2001). Computerized neurocognitive scanning: I. Methodology and validation in healthy people. Neuropsychopharmacology.

[54] Etkin A, Egner T, Peraza DM, Kandel ER, Hirsch J (2006). Resolving emotional conflict: a role for the rostral anterior cingulate cortex in modulating activity in the amygdala. Neuron.

[55] Gratton G, Coles MG, Donchin E (1992). Optimizing the use of information: strategic control of activation of responses. J. Exp. Psychol. Gen..

[56] Shen X, Tokoglu F, Papademetris X, Constable RT (2013). Groupwise whole-brain parcellation from resting-state fMRI data for network node identification. Neuroimage.

[57] Passie T, Seifert J, Schneider U, Emrich HM (2002). The pharmacology of psilocybin. Addict. Biol..

[58] Brown RT (2017). Pharmacokinetics of Escalating Doses of Oral Psilocybin in Healthy Adults. Clin. Pharmacokinet..

[59] Darmon M, Al Awabdh S, Emerit M-B, Masson J (2015). Insights into Serotonin Receptor Trafficking: Cell Membrane Targeting and Internalization. Prog. Mol. Biol. Transl. Sci..

[60] Barbey AK, Koenigs M, Grafman J (2013). Dorsolateral Prefrontal Contributions to Human Working Memory. Cortex.

[61] Jimura K, Chushak MS, Westbrook A, Braver TS (2018). Intertemporal Decision-Making Involves Prefrontal Control Mechanisms Associated with Working Memory. Cereb. Cortex.

[62] Ochsner KN, Silvers JA, Buhle JT (2012). Functional imaging studies of emotion regulation: A synthetic review and evolving model of the cognitive control of emotion. Ann. N. Y. Acad. Sci..

[63] Rive MM (2013). Neural correlates of dysfunctional emotion regulation in major depressive disorder. A systematic review of neuroimaging studies. Neurosci. Biobehav. Rev..

[64] Fales CL (2009). Antidepressant treatment normalizes hypoactivity in dorsolateral prefrontal cortex during emotional interference processing in major depression. J. Affect. Disord..

[65] Zilverstand A, Parvaz MA, Goldstein RZ (2017). Neuroimaging cognitive reappraisal in clinical populations to define neural targets for enhancing emotion regulation. A systematic review. NeuroImage.

[66] Noonan MP, Chau BKH, Rushworth MFS, Fellows LK (2017). Contrasting Effects of Medial and Lateral Orbitofrontal Cortex Lesions on Credit Assignment and Decision-Making in Humans. J. Neurosci..

[67] Elliott R, Dolan RJ, Frith CD (2000). Dissociable functions in the medial and lateral orbitofrontal cortex: evidence from human neuroimaging studies. Cereb. Cortex.

[68] Kringelbach ML, Rolls ET (2004). The functional neuroanatomy of the human orbitofrontal cortex: evidence from neuroimaging and neuropsychology. Prog. Neurobiol..

[69] Nelson JK, Reuter-Lorenz PA, Persson J, Sylvester C-YC, Jonides J (2009). Mapping interference resolution across task domains: a shared control process in left inferior frontal gyrus. Brain Res..

[70] Hassanpour MS (2018). The Insular Cortex Dynamically Maps Changes in Cardiorespiratory Interoception. Neuropsychopharmacology.

[71] Grecucci A, Giorgetta C, Bonini N, Sanfey AG (2013). Reappraising social emotions: the role of inferior frontal gyrus, temporo-parietal junction and insula in interpersonal emotion regulation. Front. Hum. Neurosci..

[72] Weiner KS, Zilles K (2016). The anatomical and functional specialization of the fusiform gyrus. Neuropsychologia.

[73] Nasr S (2011). Scene-Selective Cortical Regions in Human and Nonhuman Primates. J. Neurosci..

[74] Baker CI, Hutchison TL, Kanwisher N (2007). Does the fusiform face area contain subregions highly selective for nonfaces?. Nat. Neurosci..

[75] Grill-Spector K, Knouf N, Kanwisher N (2004). The fusiform face area subserves face perception, not generic within-category identification. Nat. Neurosci..

[76] Yovel G, Kanwisher N (2004). Face perception: domain specific, not process specific. Neuron.

[77] Roseman Leor, Demetriou Lysia, Wall Matthew B., Nutt David J., Carhart-Harris Robin L. (2018). Increased amygdala responses to emotional faces after psilocybin for treatment-resistant depression. Neuropharmacology.

[78] Drevets WC, Price JL, Furey ML (2008). Brain structural and functional abnormalities in mood disorders: implications for neurocircuitry models of depression. Brain Struct. Funct..

[79] Sheline YI (2001). Increased amygdala response to masked emotional faces in depressed subjects resolves with antidepressant treatment: an fMRI study. Biol. Psychiatry.

[80] Fu CHY (2004). Attenuation of the neural response to sad faces in major depression by antidepressant treatment: a prospective, event-related functional magnetic resonance imaging study. Arch. Gen. Psychiatry.

[81] Nichols DE (2016). Psychedelics. Pharmacol. Rev..

[82] Sampedro F (2017). Assessing the Psychedelic ‘After-Glow’ in Ayahuasca Users: Post-Acute Neurometabolic and Functional Connectivity Changes Are Associated with Enhanced Mindfulness Capacities. Int. J. Neuropsychopharmacol..

[83] Birn RM (2013). The effect of scan length on the reliability of resting-state fMRI connectivity estimates. Neuroimage.

[84] Studerus, E., Gamma, A., Kometer, M. & Vollenweider, F. X. Prediction of Psilocybin Response in Healthy Volunteers. *PLoS One* **7** (2012). 10.1371/journal.pone.0030800 PMC3281871 22363492

[85] Russ SL, Carhart-Harris RL, Maruyama G, Elliott MS (2019). States and traits related to the quality and consequences of psychedelic experiences. Psychol. Consciousness: Theory, Research, Pract..

[86] MacLean KA, Johnson MW, Griffiths RR (2011). Mystical experiences occasioned by the hallucinogen psilocybin lead to increases in the personality domain of openness. J. Psychopharmacol..

[87] Erritzoe D (2018). Effects of psilocybin therapy on personality structure. Acta Psychiatr. Scand..

[88] Griffiths R, Richards W, Johnson M, McCann U, Jesse R (2008). Mystical-type experiences occasioned by psilocybin mediate the attribution of personal meaning and spiritual significance 14 months later. J. Psychopharmacol..

[89] Griffiths RR (2011). Psilocybin occasioned mystical-type experiences: immediate and persisting dose-related effects. Psychopharmacol..

[90] Nardou R (2019). Oxytocin-dependent reopening of a social reward learning critical period with MDMA. Nat..

[91] Johnson M, Richards W, Griffiths R (2008). Human hallucinogen research: guidelines for safety. J. Psychopharmacol..

[92] Griffiths, R. R., Richards, W. A., McCann, U. & Jesse, R. Psilocybin can occasion mystical-type experiences having substantial and sustained personal meaning and spiritual significance. *Psychopharmacology (Berl.)* **187**, 268–283; discussion 284–292 (2006). 10.1007/s00213-006-0457-5 16826400

[93] Griffiths RR (2018). Psilocybin-occasioned mystical-type experience in combination with meditation and other spiritual practices produces enduring positive changes in psychological functioning and in trait measures of prosocial attitudes and behaviors. J. Psychopharmacol..

[94] Barrett Frederick S., Carbonaro Theresa M., Hurwitz Ethan, Johnson Matthew W., Griffiths Roland R. (2018). Double-blind comparison of the two hallucinogens psilocybin and dextromethorphan: effects on cognition. Psychopharmacology.

[95] Carbonaro TM, Johnson MW, Hurwitz E, Griffiths RR (2018). Double-blind comparison of the two hallucinogens psilocybin and dextromethorphan: similarities and differences in subjective experiences. Psychopharmacol..

[96] Tottenham N (2009). The NimStim set of facial expressions: judgments from untrained research participants. Psychiatry Res..

[97] Gur RC (2002). Brain activation during facial emotion processing. Neuroimage.

[98] Gur RC (2010). A cognitive neuroscience-based computerized battery for efficient measurement of individual differences: standardization and initial construct validation. J. Neurosci. Methods.

[99] Wager TD, Nichols TE (2003). Optimization of experimental design in fMRI: a general framework using a genetic algorithm. Neuroimage.

[100] Bradley, M. M. & Lang, P. J. *Affective Norms for English Words (ANEW): Instruction Manual and Affective Ratings*. (1999).

[101] Tukey JW (1949). Comparing Individual Means in the Analysis of Variance. Biometrics.

[102] Calhoun VD (2017). The impact of T1 versus EPI spatial normalization templates for fMRI data analyses. Hum. Brain Mapp..

[103] Whitfield-Gabrieli S, Nieto-Castanon A (2012). Conn: a functional connectivity toolbox for correlated and anticorrelated brain networks. Brain Connect..

[104] Holm S (1979). A Simple Sequentially Rejective Multiple Test Procedure. Scand. J. Stat..

[105] Lindquist MA, Geuter S, Wager TD, Caffo BS (2019). Modular preprocessing pipelines can reintroduce artifacts into fMRI data. Hum. Brain Mapp..

[106] Hallquist MN, Hwang K, Luna B (2013). The nuisance of nuisance regression: spectral misspecification in a common approach to resting-state fMRI preprocessing reintroduces noise and obscures functional connectivity. Neuroimage.

