# Supplementary material for: Emotions and brain function are altered up to one month after a single high dose of psilocybin
Source: Sci Rep. 2020 Feb 10;10:2214. doi: 10.1038/s41598-020-59282-y (PMC7010702; doi:10.1038/s41598-020-59282-y)
Supplement: Supplementary file 1 — Supplementary Information. [file 41598_2020_59282_MOESM1_ESM.pdf]

Supplementary Information

Emotions and brain function are altered up to one month after a single high dose of psilocybin

Frederick S. Barrett, Ph.D.[1], Manoj K. Doss, Ph.D.[1], Nathan D. Sepeda, B.S.[1], James J. Pekar, Ph.D.[2,3], Roland R. Griffiths, Ph.D[1,4].

[1]Department of Psychiatry and Behavioral Sciences, Johns Hopkins University School of Medicine, Baltimore, MD, 21224, USA. [2]F.M. Kirby Research Center for Functional Brain Imaging, Kennedy Krieger Institute, Baltimore, MD, 21205, USA. [3]Russell H. Morgan Department of Radiology and Radiological Science, Johns Hopkins University, Baltimore, MD, 21205, USA. [4]Department of Neuroscience, Johns Hopkins University School of Medicine, Baltimore, MD, 21224, USA.

Table S1. Descriptive statistics for self-report measures

	Baseline	1 week	1 month
Measure	Mean (*SE*)	Mean (*SE*)	Mean (*SE*)
Depression (DASS)	1.82 (0.57)	1.09 (0.73)	1.64 (0.59)
Anxiety (DASS)	1.64 (1.07)	1.64 (0.75)	1.45 (0.90)
Stress (DASS)	4.91 (1.06)	2.00 (0.60)	3.27 (1.12)
Joy (DPES)	5.45 (0.21)	6.02 (0.17)	5.92 (0.21)
Content (DPES)	5.62 (0.24)	6.27 (0.16)	6.13 (0.23)
Pride (DPES)	5.42 (0.22)	5.96 (0.14)	5.95 (0.18)
Love (DPES)	5.61 (0.17)	5.92 (0.25)	5.98 (0.17)
Compassion (DPES)	5.75 (0.31)	6.25 (0.26)	6.00 (0.30)
Amusement (DPES)	5.02 (0.31)	5.60 (0.32)	5.71 (0.31)
Awe (DPES)	5.52 (0.26)	6.06 (0.22)	5.88 (0.24)
Positive Affect (PANAS-X)	38.18 (1.41)	40.64 (1.28)	40.18 (1.92)
Negative Affect (PANAS-X)	16.09 (1.00)	12.36 (0.93)	16.09 (0.80)
Tension (POMS)	4.82 (1.22)	1.73 (0.49)	3.82 (1.19)
Depression (POMS)	3.36 (0.81)	0.55 (0.25)	2.64 (1.03)
Anger (POMS)	4.91 (1.34)	2.18 (0.58)	4.64 (0.98)
Fatigue (POMS)	4.09 (1.37)	2.27 (1.21)	2.72 (1.06)
Confusion (POMS)	5.55 (1.19)	4.27 (0.79)	4.72 (1.21)
Vigor (POMS)	19.09 (1.35)	21.45 (1.77)	22.09 (2.15)
Total Mood Disturbance (POMS)	3.64 (5.67)	-10.5 (3.23)	-3.55 (6.19)
State Anxiety (STAI)	28.0 (2.41)	22.82 (1.37)	25.64 (2.56)
Trait Anxiety (STAI)	31.36 (1.91)	28.55 (1.32)	27.55 (1.95)
Extraversion (BFI)	3.46 (0.190)		3.69 (0.183)
Agreeableness (BFI)	4.43 (0.099)		4.33 (0.117)
Conscientiousness (BFI)	3.82 (0.172)		3.98 (0.159)
Neuroticism (BFI)	1.82 (0.213)		1.67 (0.175)
Openness (BFI)	4.17 (0.129)		4.22 (0.151)
Absorption (TAS)	0.98 (0.176)		1.31 (0.233)

Individual participant data for amygdala response to emotional stimuli

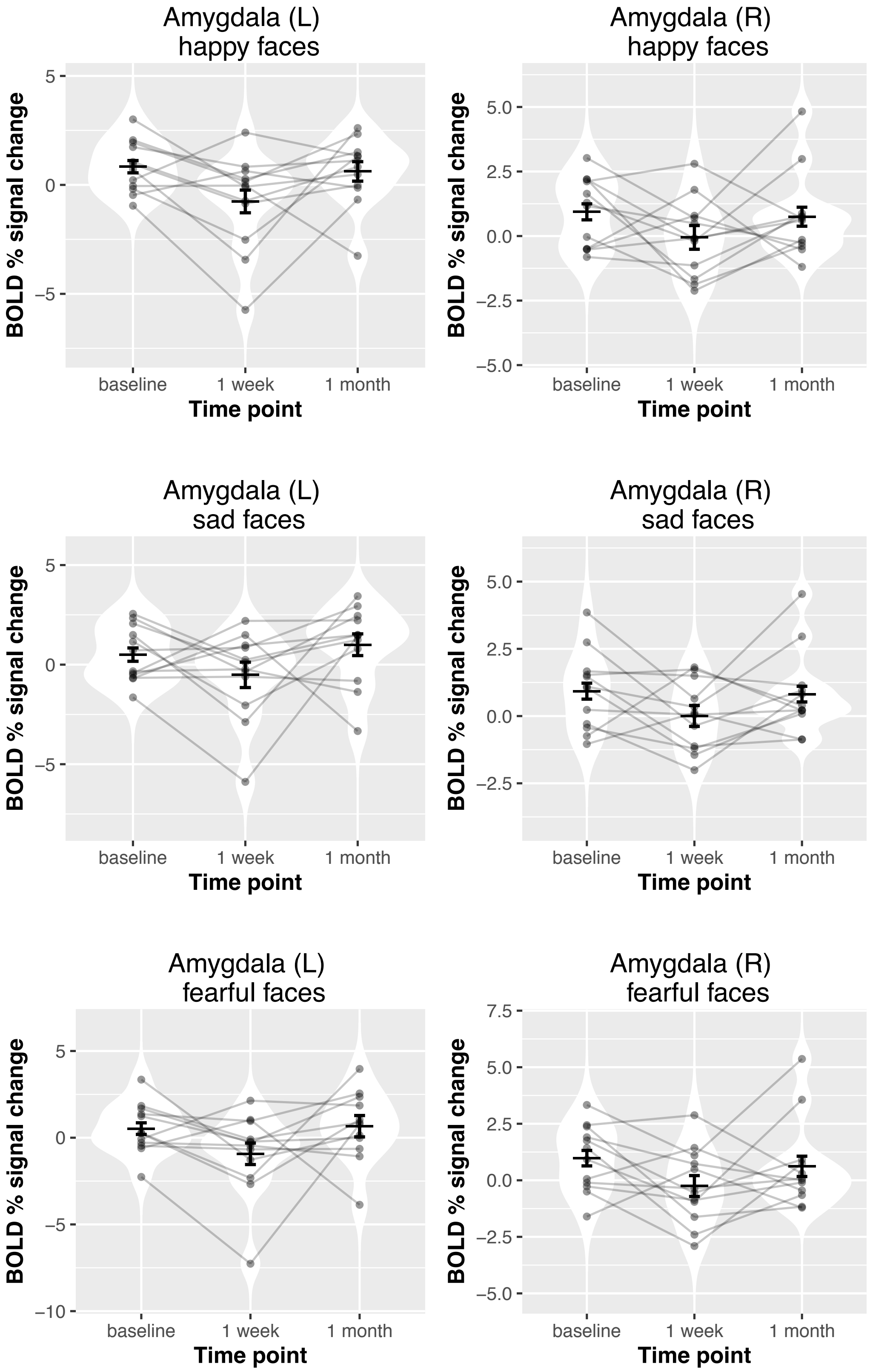

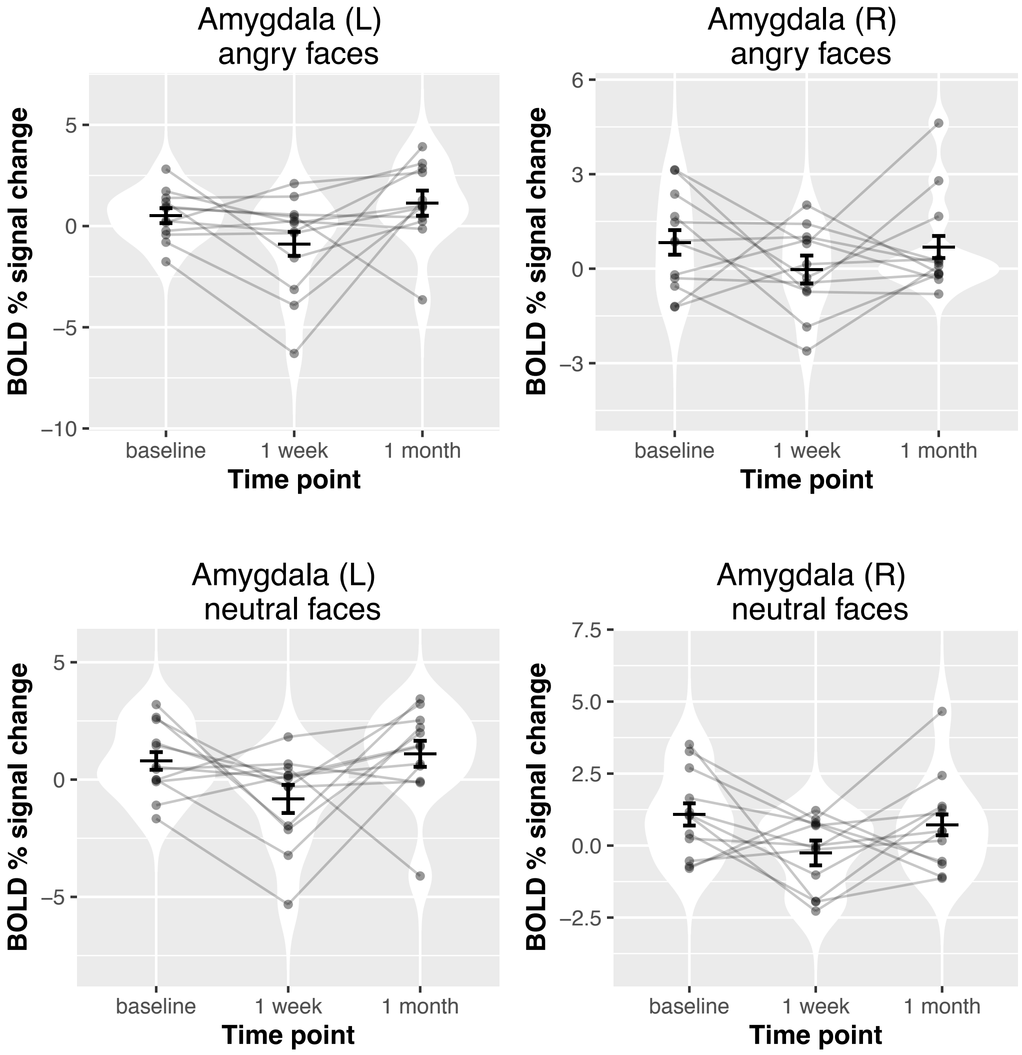

Figure S1. Subject-level data showing amygdala response to emotional stimuli. Each panel displays a set of violin plots that depict the change in BOLD % signal change within each subject for the [emotion > all stimuli] contrast for the given region of interest (left or right amygdala) and the given emotional category (happy, sad, fearful, angry, and neutral faces). Means at each timepoint (baseline, 1 week post-psilocybin, and 1 month post-psilocybin) and standard error are plotted as black horizontal lines and error bars, and the approximate distribution of values is presented in the violin. Individual contrast values for each region, time point, and subject are plotted as points, and the points for each subject are connected by a solid gray line.

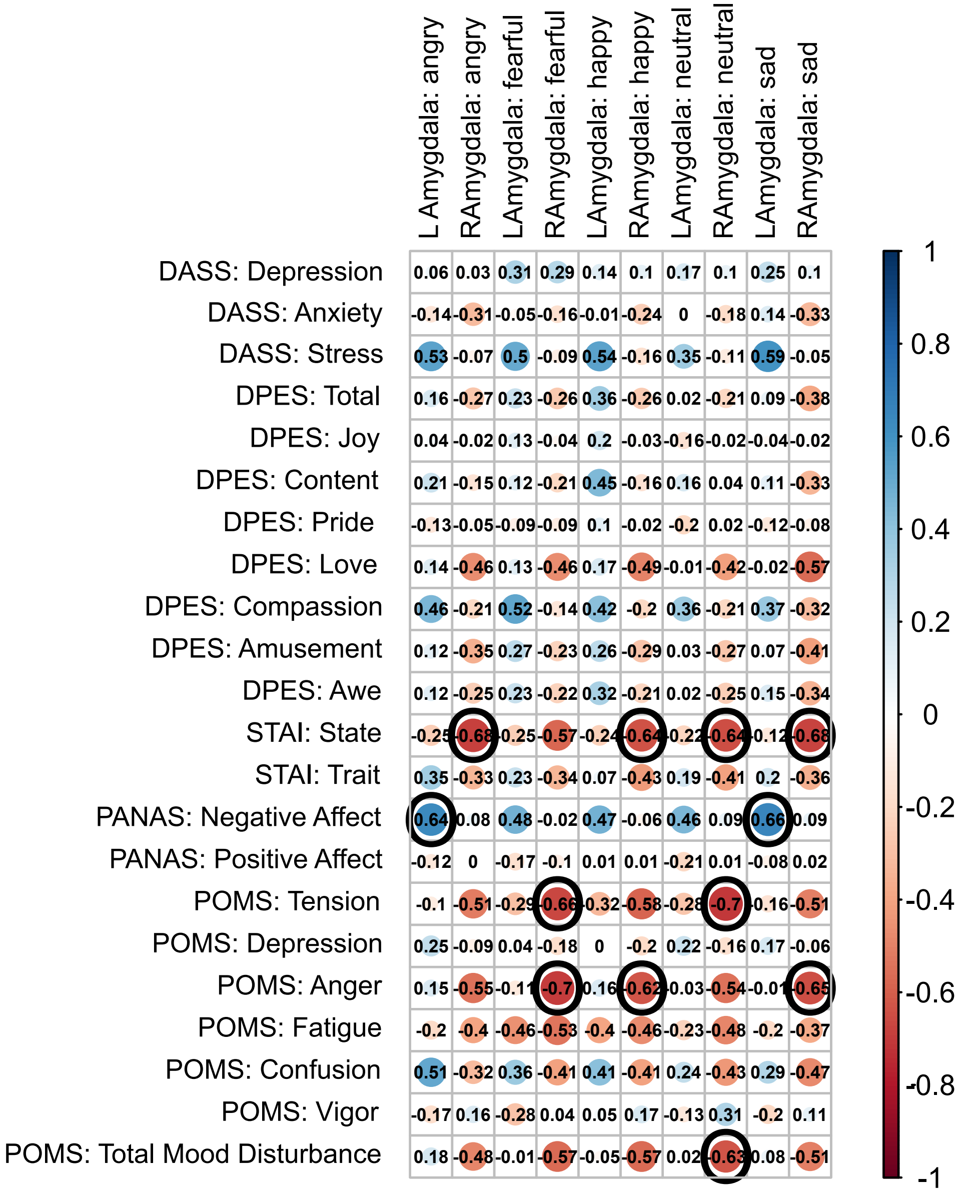

Figure S2. Associations between changes in self-report affect and changes in amygdala response in the emotion recognition task between baseline and 1 week post-psilocybin. Each cell of the figure presents the strength of Pearson correlation between change in the self-report measure on the vertical axis and change in amygdala response (left or right) to a given facial affect category on the horizontal axis. Correlations that are significant ($\alpha = 0.05$, uncorrected) are circled. DASS: Depression, Anxiety, and Stress Scale; DPES: Dispositional Positive Emotion Scale; STAI: State-Trait Anxiety Inventory; PANAS: Positive and Negative Affect Scale; POMS: Profile of Mood States.

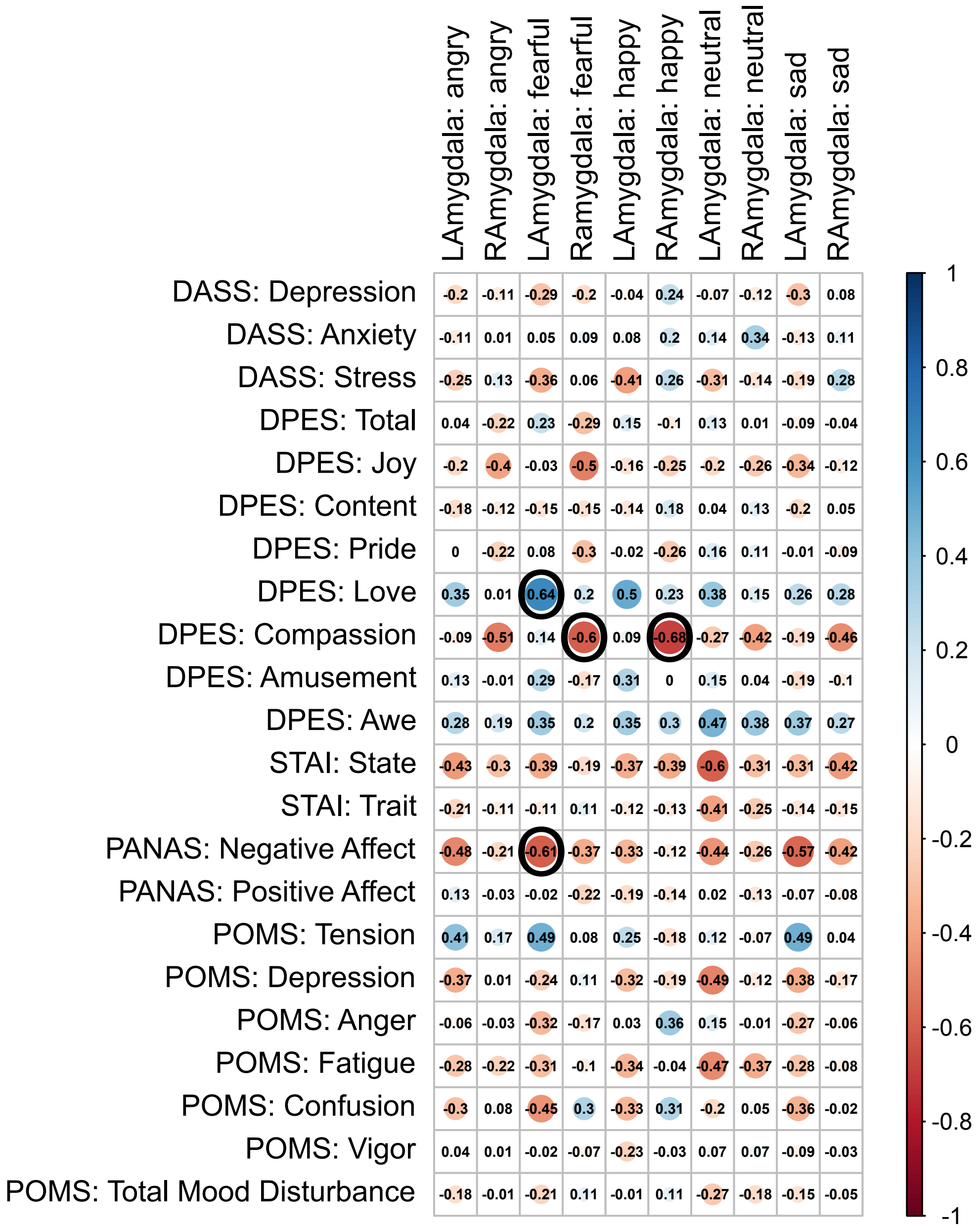

Figure S3. Associations between changes in self-report affect and changes in amygdala response in the emotion recognition task between baseline and 1 month post-psilocybin. Each cell of the figure presents the strength of Pearson correlation between change in the self-report measure on the vertical axis and change in amygdala response (left or right) to a given facial affect category on the horizontal axis. Correlations that are significant (α = 0.05, uncorrected) are circled. DASS: Depression, Anxiety, and Stress Scale; DPES: Dispositional Positive Emotion Scale; STAI: State-Trait Anxiety Inventory; PANAS: Positive and Negative Affect Scale; POMS: Profile of Mood States.

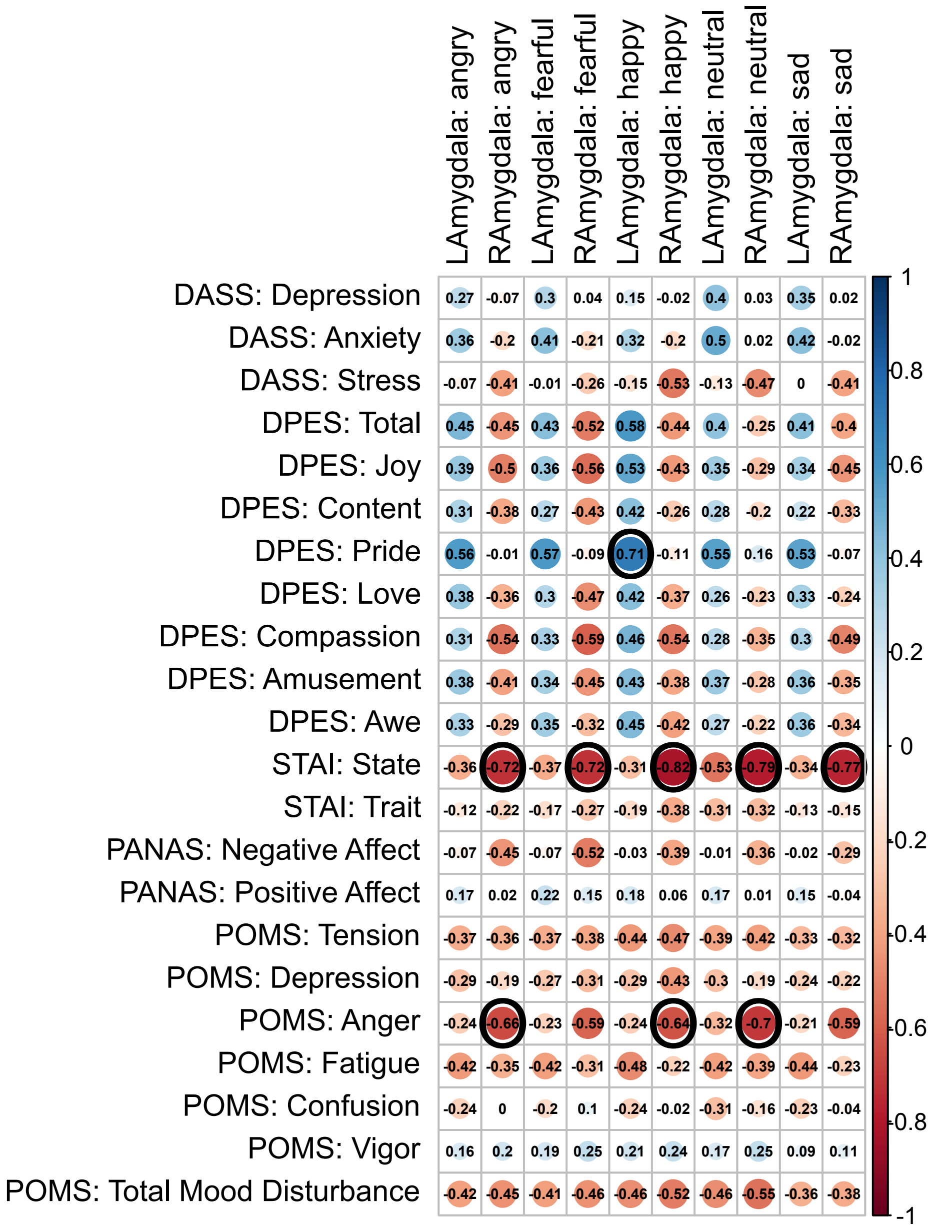

Figure S4. Associations between changes in self-report affect and changes in amygdala response in the emotion recognition task between 1 week and 1 month post-psilocybin. Each cell of the figure presents the strength of Pearson correlation between change in the self-report measure on the vertical axis and change in amygdala response (left or right) to a given facial affect category on the horizontal axis. Correlations that are significant (α = 0.05, uncorrected) are circled. DASS: Depression, Anxiety, and Stress Scale; DPES: Dispositional Positive Emotion Scale; STAI: State-Trait Anxiety Inventory; PANAS: Positive and Negative Affect Scale; POMS: Profile of Mood States.

Resting State Dynamic Functional Connectivity

Dynamic functional connectivity of every edge was computed for each participant using dynamic conditional correlations (DCC)[1]. The variance of each edge's DCC time series was then calculated. Compared to the commonly used sliding window approach, DCCs have been shown to be more reliable and more sensitive to dynamic changes in correlations, and do not suffer from artifacts introduced by arbitrary windowing practices[2]. There were no observable differences between time points in the variance of the dynamic functional connectivity time course in edge-wise comparisons (Figure S2). However, there were numerical increases in dynamic functional connectivity of the default mode network (DM) to fronto-parietal (FP) and medial frontal (MF) networks at one week post-psilocybin compared to baseline, further numerical increases in dynamic functional connectivity of the DM to most other networks at one month post-psilocybin compared to baseline, with an overall decrease in dynamic functional connectivity among the remaining networks at one week and one month post-psilocybin, and overall greater functional connectivity of DM and MedV networks to most other networks at 1 week than at 1 month post-psilocybin (Figure S3).

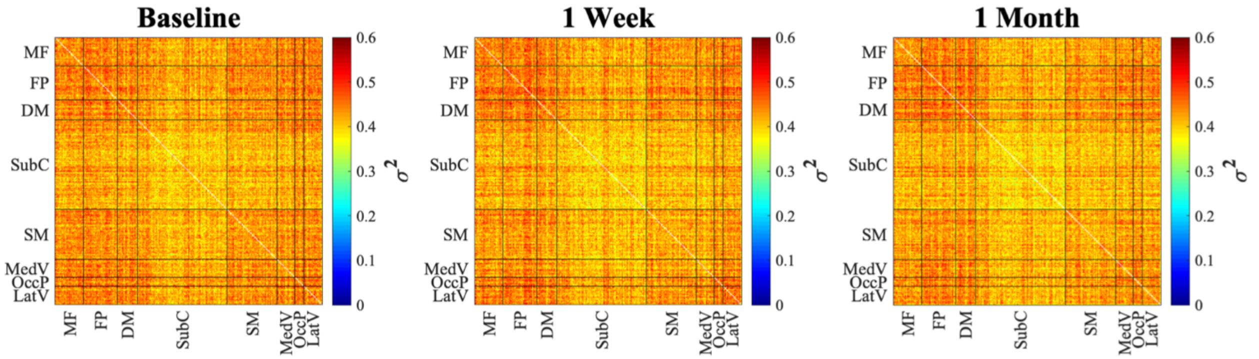

Figure S5. Variance in the time course of dynamic functional connectivity for all pairwise functional connections (268 nodes × 268 nodes = 35,778 edges) at each time point. Each row and each column represents a single node (ROI) as defined by the Shen 268-node functional brain

atlas[3]. The color of each off-diagonal cell represents the variance in the time course of dynamic functional connectivity in each given edge (each between-node connection). Nodes are grouped together in rows and columns by network as defined in the Shen atlas, with black lines marking the border between networks in the matrix. MF = medial frontal network, FP = frontoparietal network, DM = default mode network, SubC = subcortical-cerebellum network (including the salience network), SM = somatosensory-motor network, MedV = medial visual network, OccP = occipital pole network, and LatV = lateral visual network.

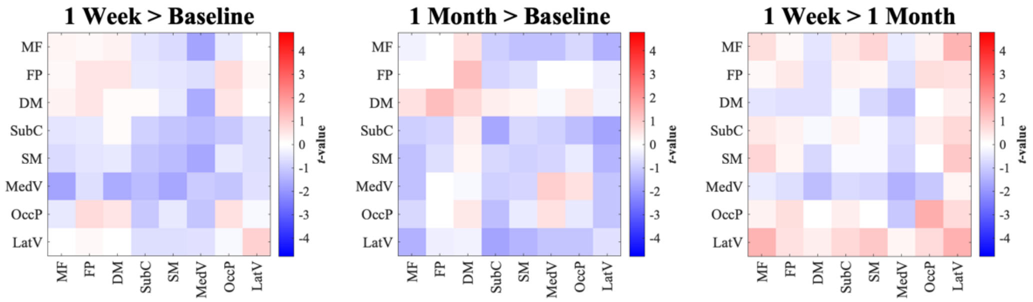

Figure S6. Differences in the variance of the time course of dynamic functional connectivity within and between canonical networks for 1 Week > Baseline, 1 Month > Baseline and 1 Week > 1 Month. Each row and column represents a single brain network as defined by the Shen 268-node functional brain atlas[3]. The diagonal cells represent differences between time points in the average variance across edges within a network, and off-diagonal cells represent average differences in the variance of between-network edges. MF = medial frontal network, FP = frontoparietal network, DM = default mode network, SubC = subcortical-cerebellum network (including the salience network), SM = somatosensory-motor network, MedV = medial visual network, OccP = occipital pole network, and LatV = lateral visual network.

Entropy of Resting State Dynamic Functional Connectivity

The entropy of each edge was computed for every participant by first discretizing the dynamic correlation time series via binning and then calculating the information entropy

corrected by bin width. Because binning can be somewhat arbitrary and produce different results at different levels of binnng, entropy was calculated separately using bin widths in a range of 10 to 60 TRs in steps of 10. The pattern of results did not differ by bin width. There were no observed differences between time points in the entropy of dynamic functional connectivity in edge-wise (Figure S4) comparisons. The pattern of numerical change in entropy of dynamic functional connectivity between networks (Figure S5) was similar to the pattern of numerical increases in the variance in dynamic functional connectivity (Figure S3), with slight increases in entropy of the dynamic functional connectivity of DM to FP and MF edges at one week post-psilocybin compared to baseline, further increases in the entropy of functional connectivity of the DM to most other networks at one month post-psilocybin compared to baseline, with an overall decrease in the entropy of dynamic functional connectivity among the remaining networks at one week and one month post-psilocybin compared to baseline, and overall greater the entropy of dynamic functional connectivity of DM and MedV networks to most other networks at 1 week than at 1 month post-psilocybin (Figure S5).

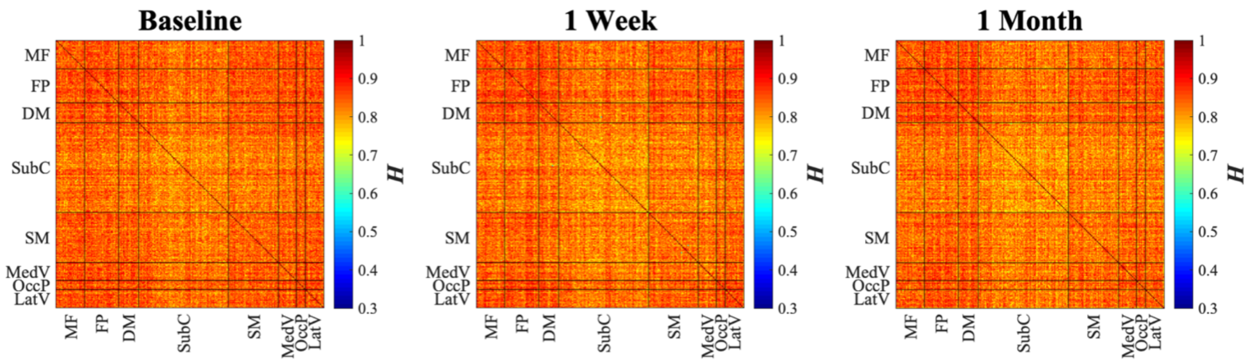

Figure S7. Differences in the entropy (*H*) of dynamic functional connectivity for all pairwise functional connections (268 nodes × 268 nodes = 35,778 edges) at each time point. Each row and each column represents a single node (ROI) as defined by the Shen 268-node functional brain atlas[3]. The color of each off-diagonal cell represents the entropy of dynamic functional

connectivity in each given edge (each between-node connection). Nodes are grouped together in rows and columns by network as defined in the Shen atlas, with black lines marking the border between networks in the matrix. MF = medial frontal network, FP = frontoparietal network, DM = default mode network, SubC = subcortical-cerebellum network (including the salience network), SM = somatosensory-motor network, MedV = medial visual network, OccP = occipital pole network, and LatV = lateral visual network.

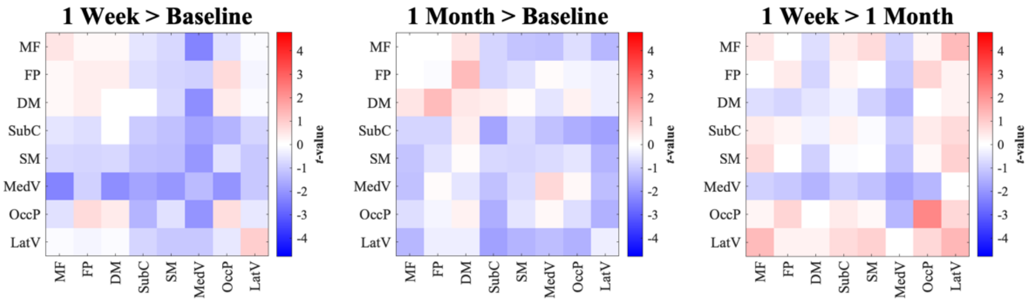

Figure S8. Differences in the entropy of dynamic functional connectivity within and between canonical networks for 1 Week > Baseline, 1 Month > Baseline, 1 Week > 1 Month. Each row and column represents a single brain network as defined by the Shen 268-node functional brain atlas[3]. The diagonal represents differences between time points in the average entropy of dynamic functional connectivity across edges within a network, and off-diagonal cells represent average differences in the entropy of dynamic functional connectivity of between-network edges. MF = medial frontal network, FP = frontoparietal network, DM = default mode network, SubC = subcortical-cerebellum network (including the salience network), SM = somatosensory-motor network, MedV = medial visual network, OccP = occipital pole network, and LatV = lateral visual network.

1. Lindquist, M. A., Xu, Y., Nebel, M. B. & Caffo, B. S. Evaluating Dynamic Bivariate Correlations in Resting-state fMRI: A comparison study and a new approach. *Neuroimage* **101**, 531–546 (2014).
2. Choe, A. S. *et al.* Comparing test-retest reliability of dynamic functional connectivity methods. *Neuroimage* **158**, 155–175 (2017).
3. Shen, X., Tokoglu, F., Papademetris, X. & Constable, R. T. Groupwise whole-brain parcellation from resting-state fMRI data for network node identification. *Neuroimage* **82**, 403–415 (2013).
